# Supplementary material for: Genome-Wide Classification and Phylogenetic Analyses of the GDSL-Type Esterase/Lipase (GELP) Family in Flowering Plants
Source: Int J Mol Sci. 2022 Oct 11;23(20):12114. doi: 10.3390/ijms232012114 (PMC9602515; doi:10.3390/ijms232012114)
Supplement: Supplementary file 1 [file ijms-23-12114-s001.zip › Figure S1.pptx]

## Slide 1
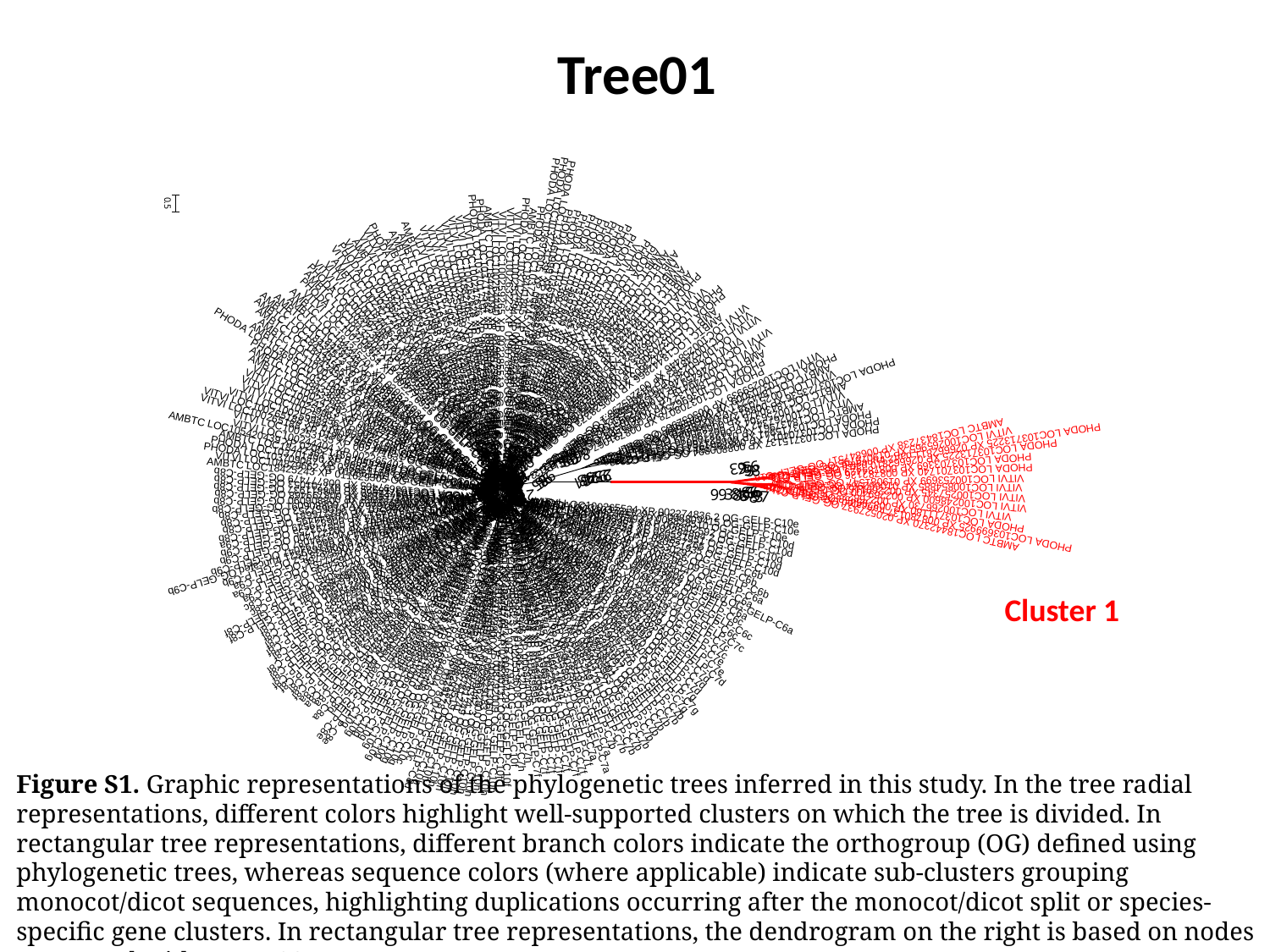

Tree01
Cluster 1
Figure S1. Graphic representations of the phylogenetic trees inferred in this study. In the tree radial representations, different colors highlight well-supported clusters on which the tree is divided. In rectangular tree representations, different branch colors indicate the orthogroup (OG) defined using phylogenetic trees, whereas sequence colors (where applicable) indicate sub-clusters grouping monocot/dicot sequences, highlighting duplications occurring after the monocot/dicot split or species-specific gene clusters. In rectangular tree representations, the dendrogram on the right is based on nodes supported with aLRT>90.

## Slide 2
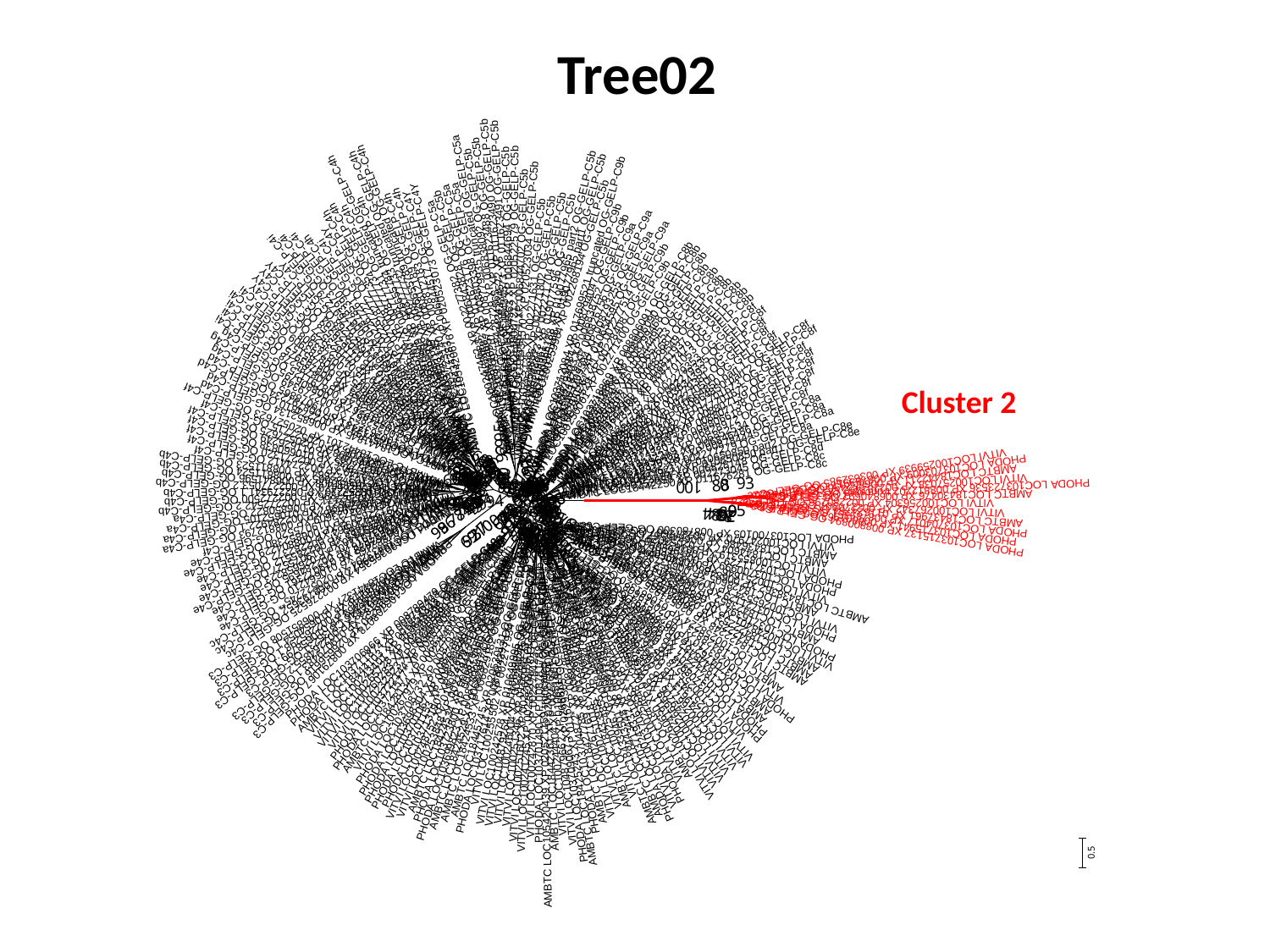

Tree02
Cluster 2

## Slide 3
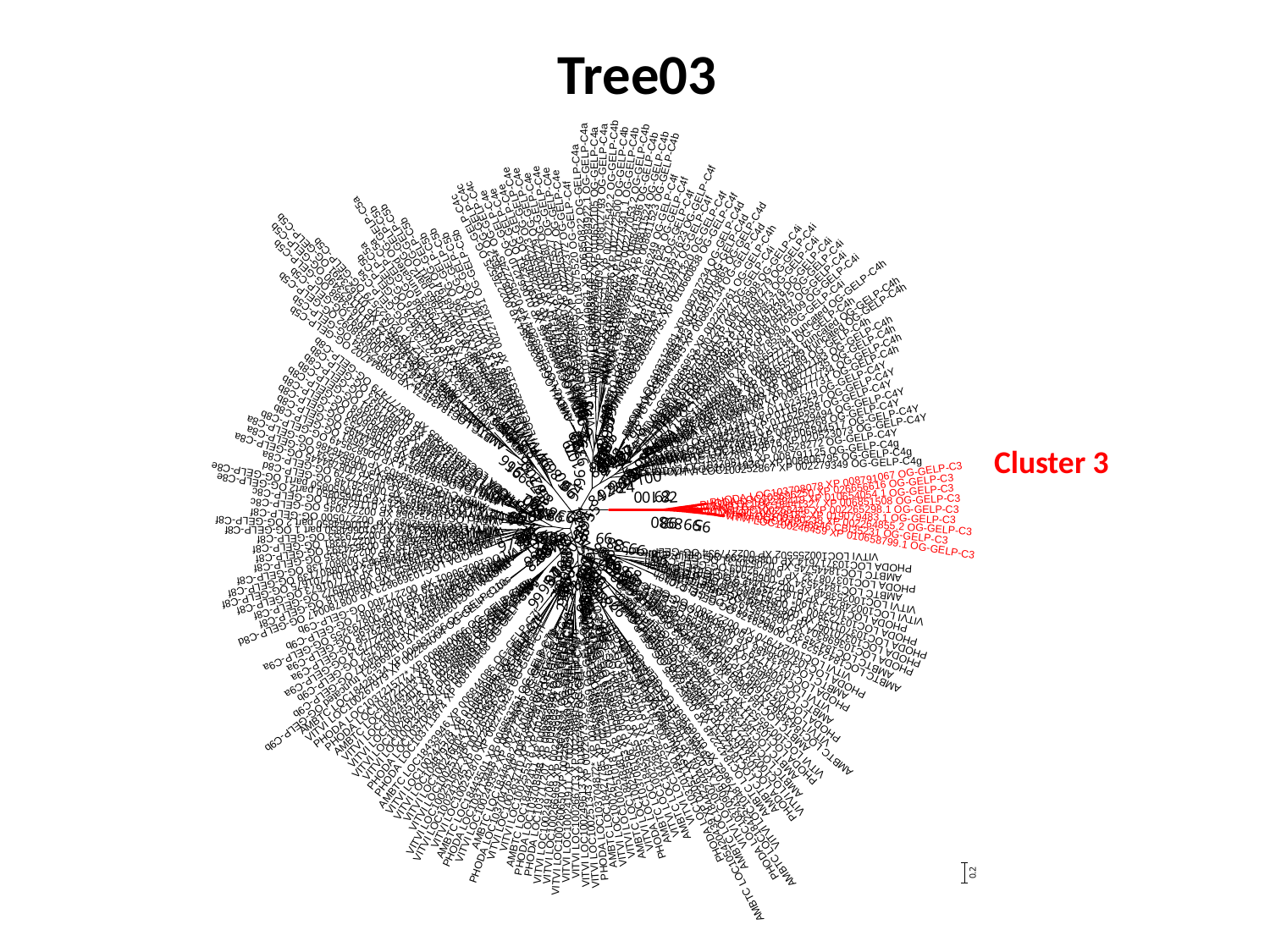

Tree03
Cluster 3

## Slide 4
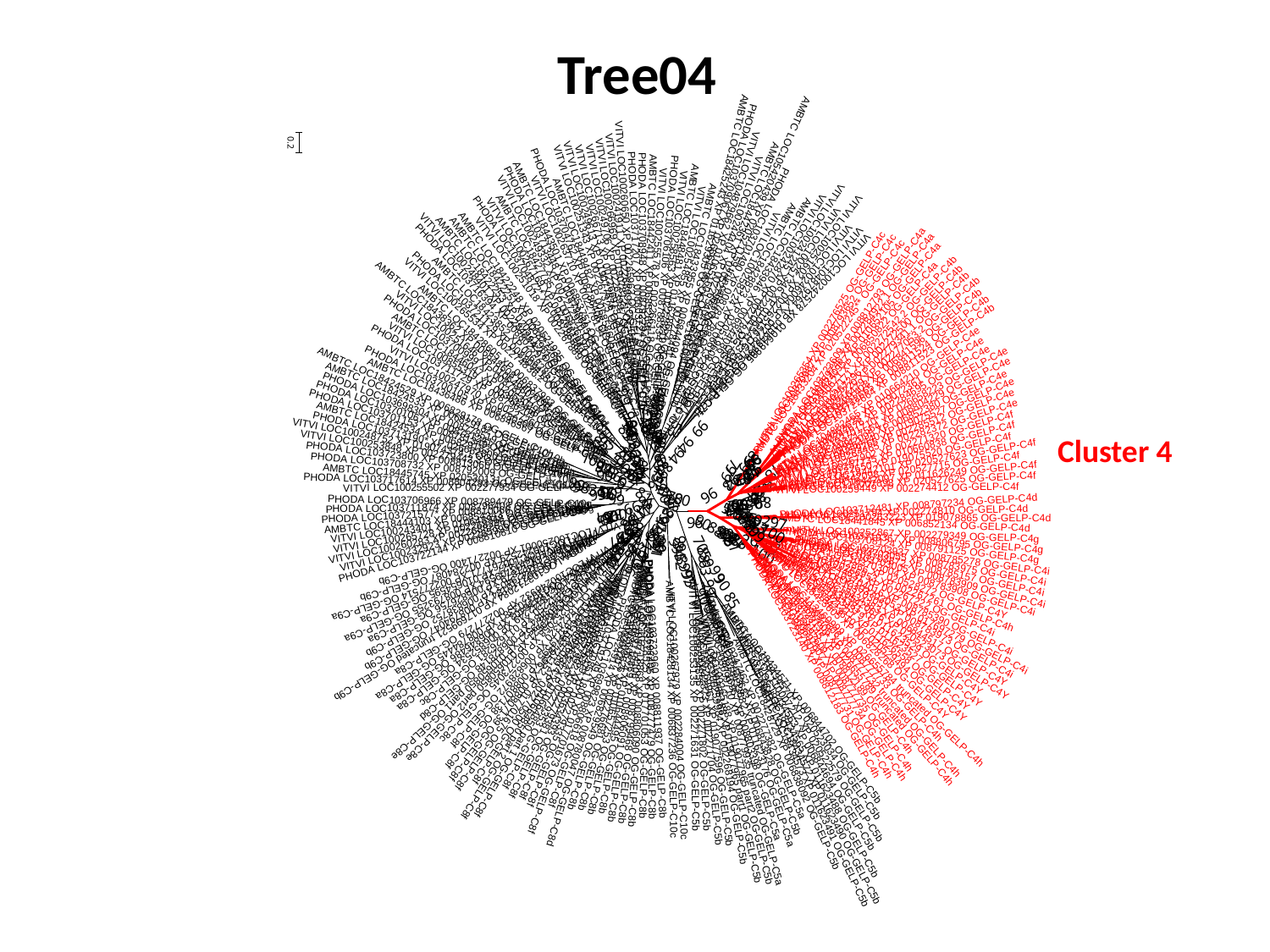

Tree04
Cluster 4

## Slide 5
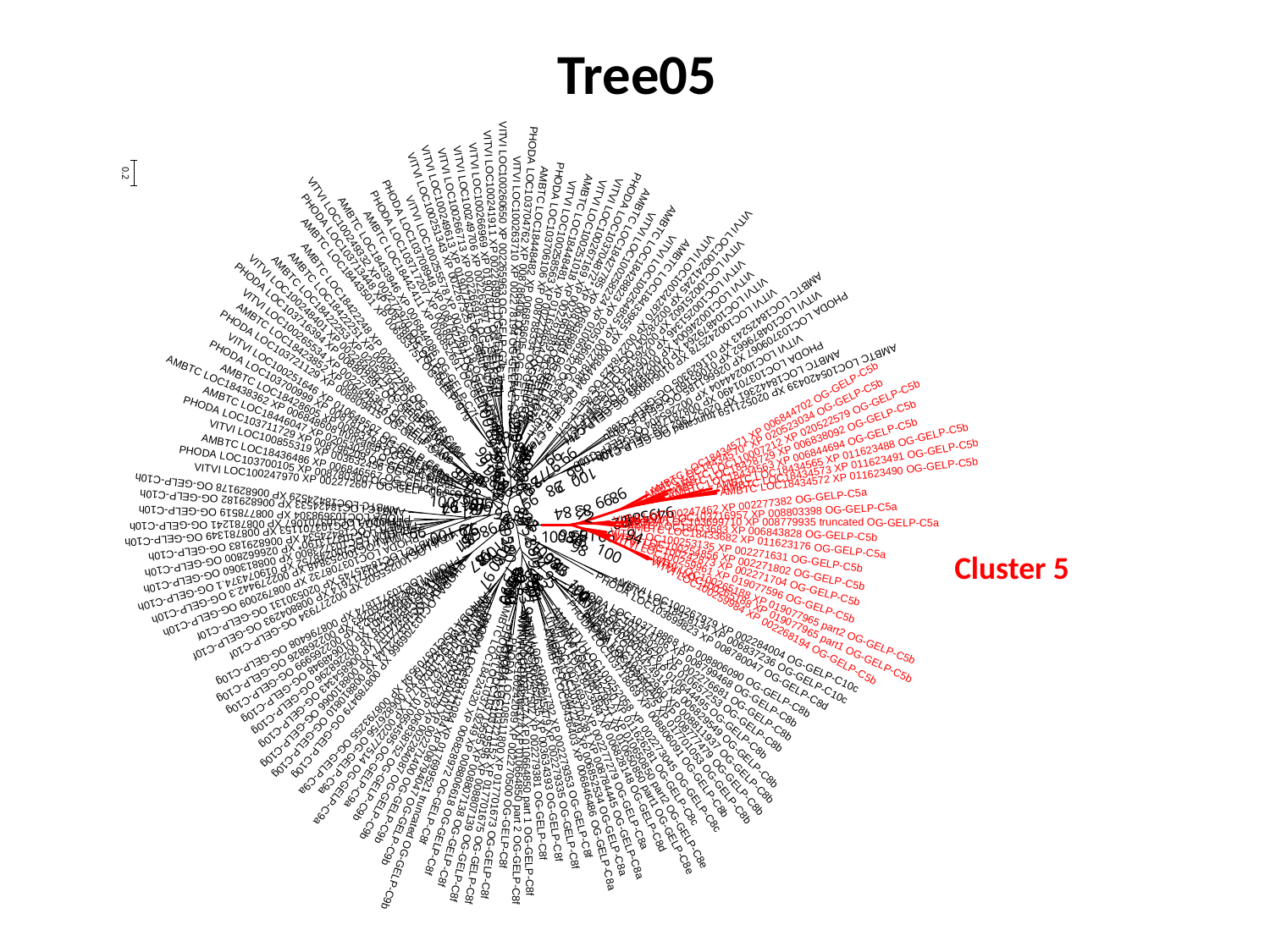

Tree05
Cluster 5

## Slide 6
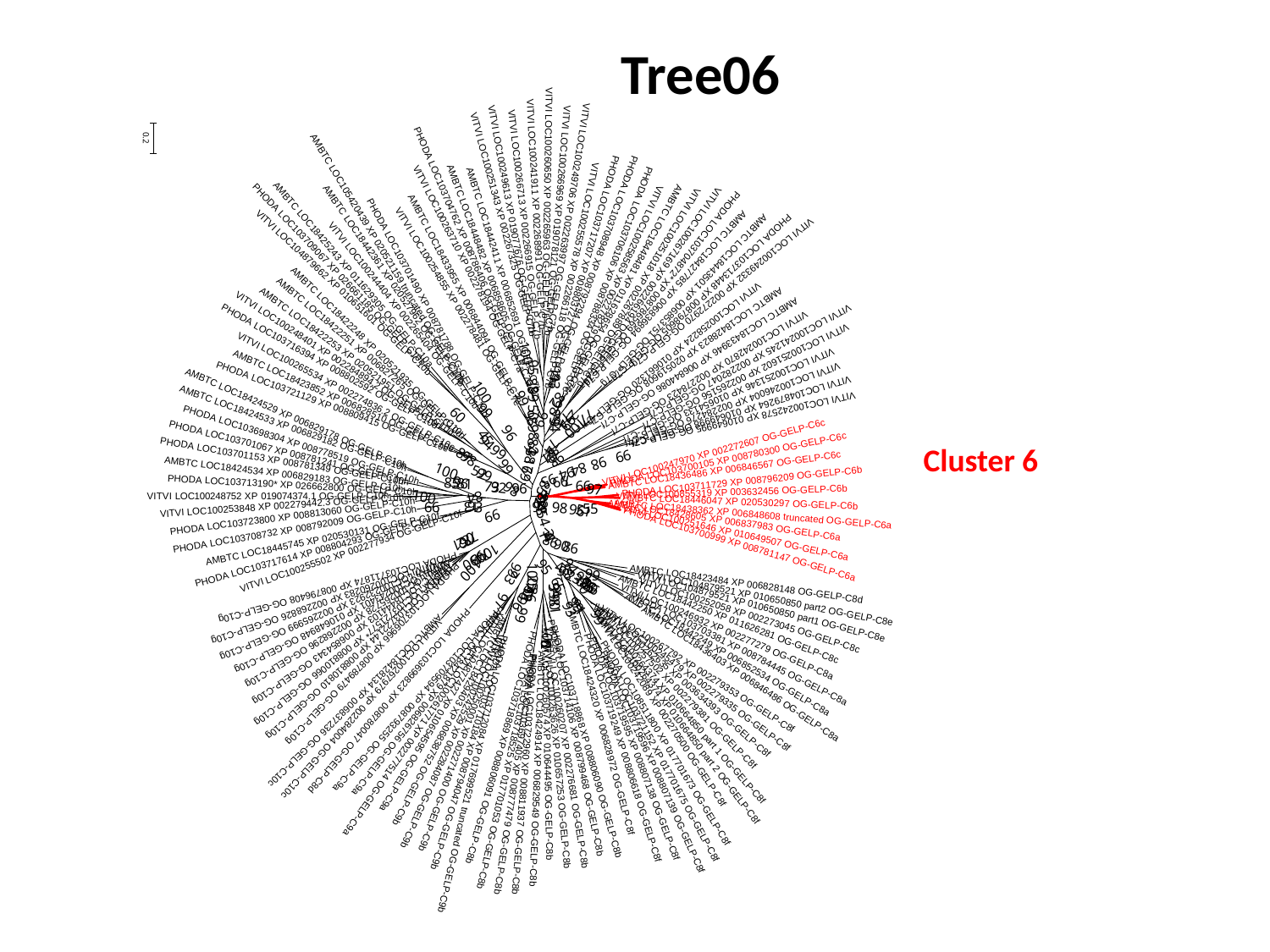

Tree06
Cluster 6

## Slide 7
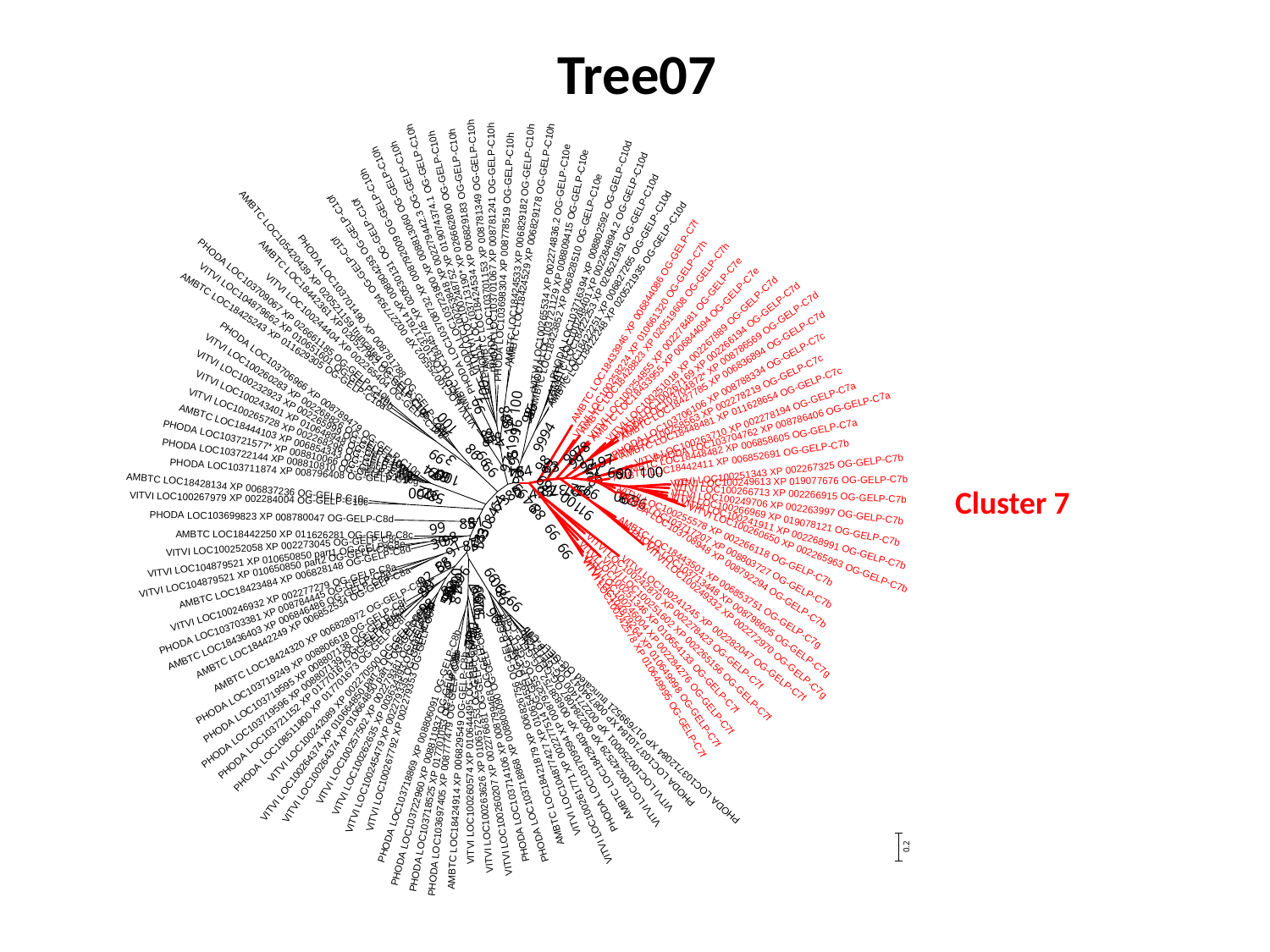

Tree07
Cluster 7

## Slide 8
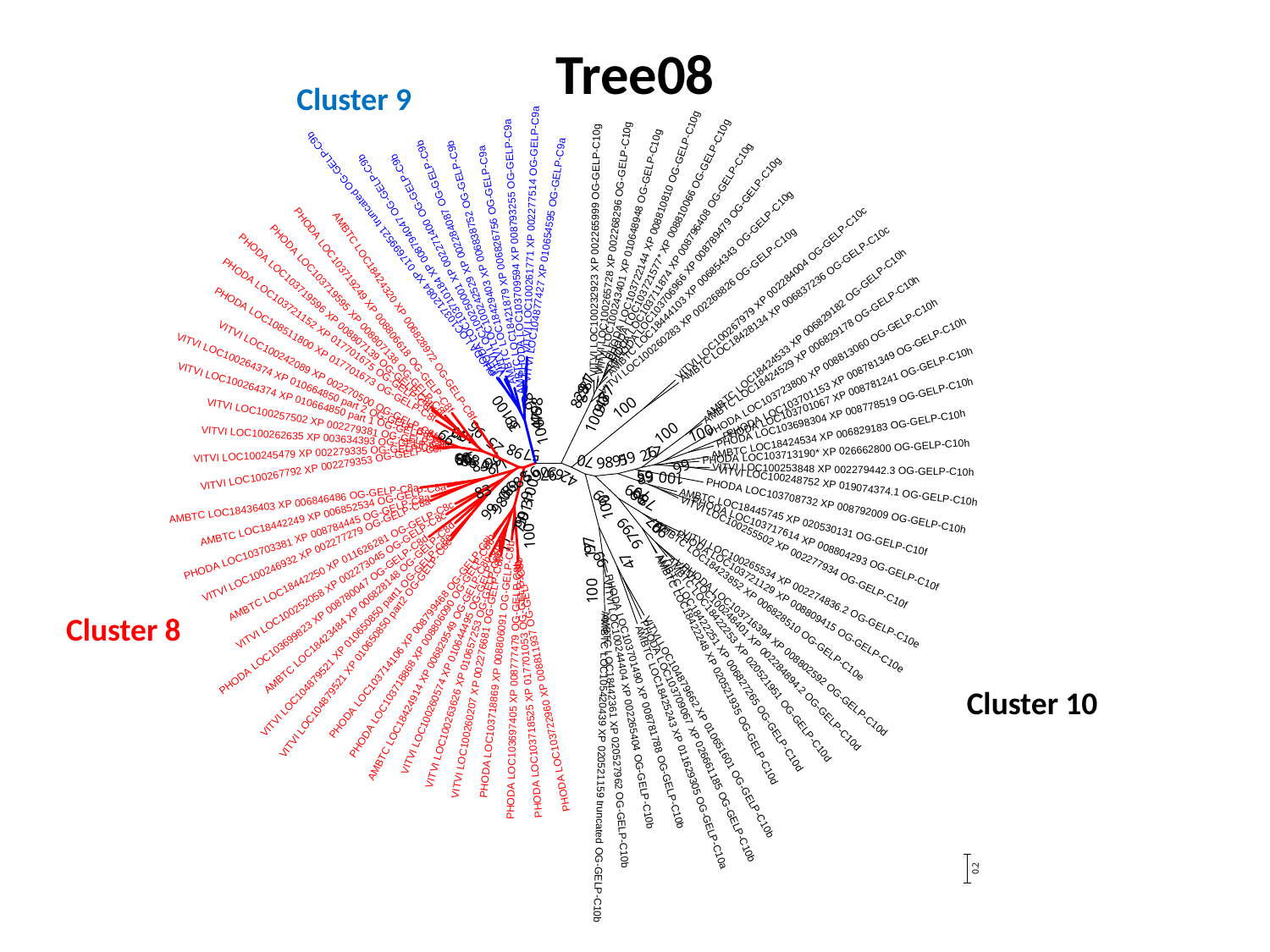

Tree08
Cluster 9
Cluster 8
Cluster 10

## Slide 9
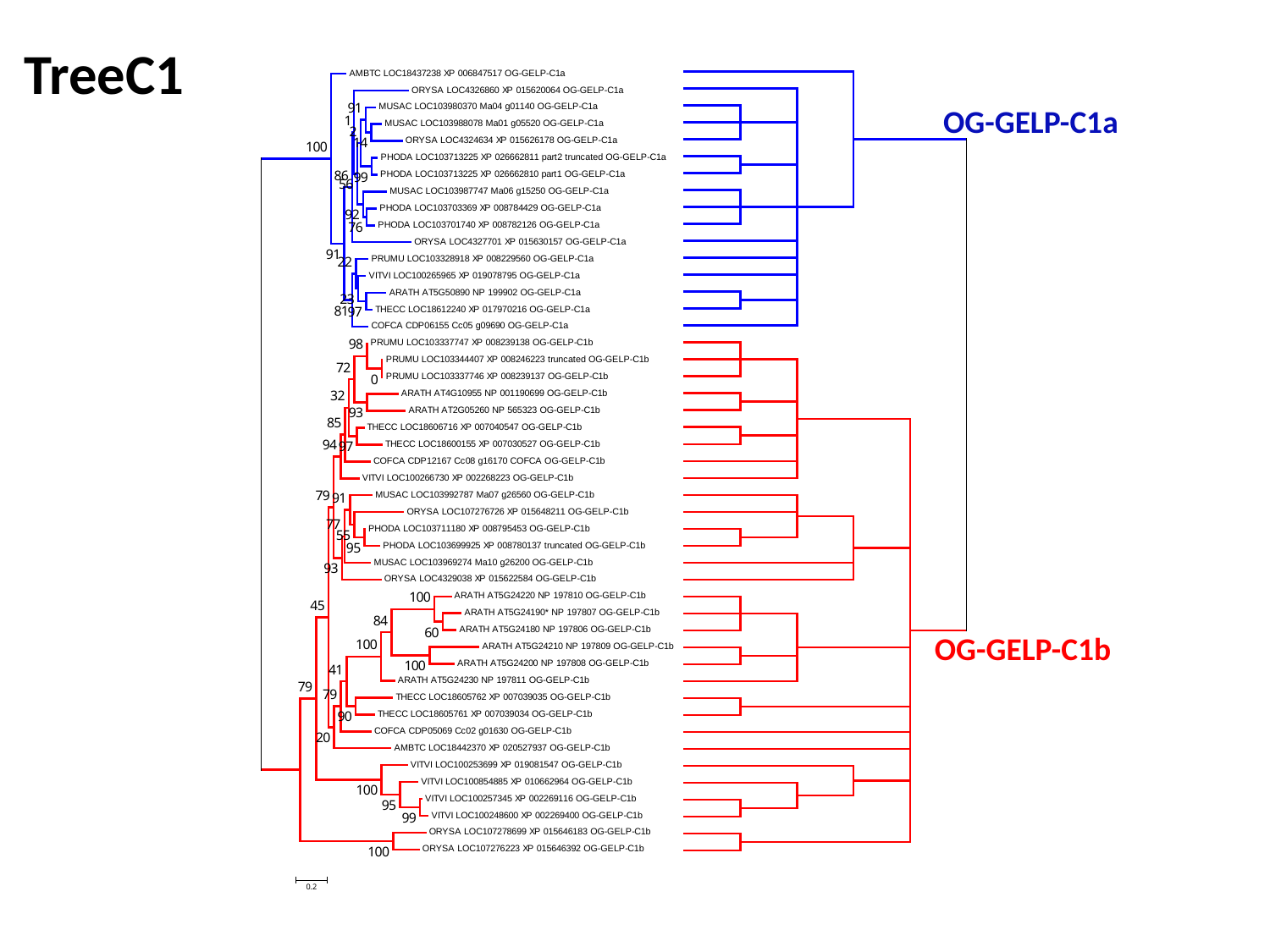

TreeC1
OG-GELP-C1a
OG-GELP-C1b

## Slide 10
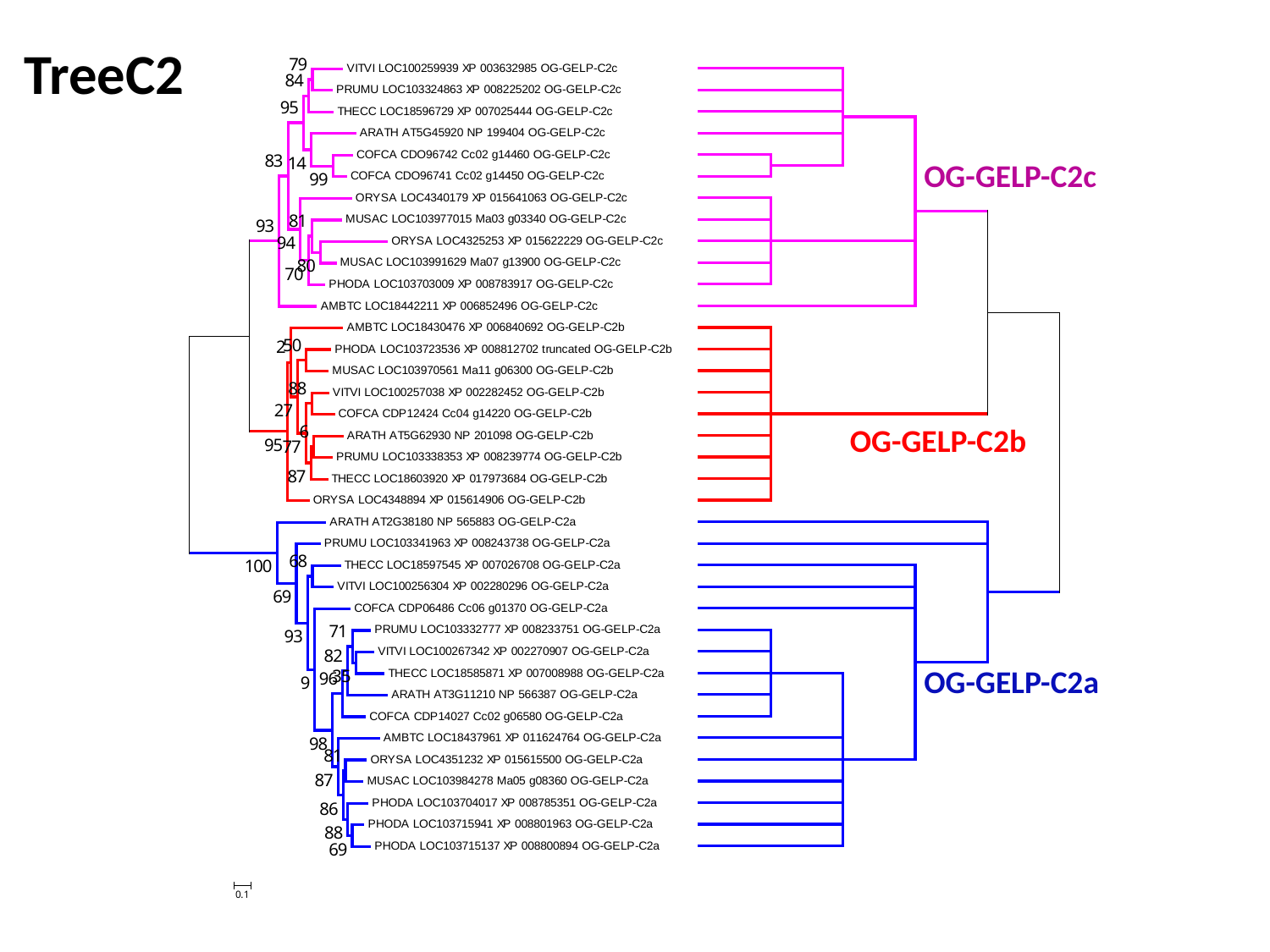

TreeC2
OG-GELP-C2c
OG-GELP-C2b
OG-GELP-C2a

## Slide 11
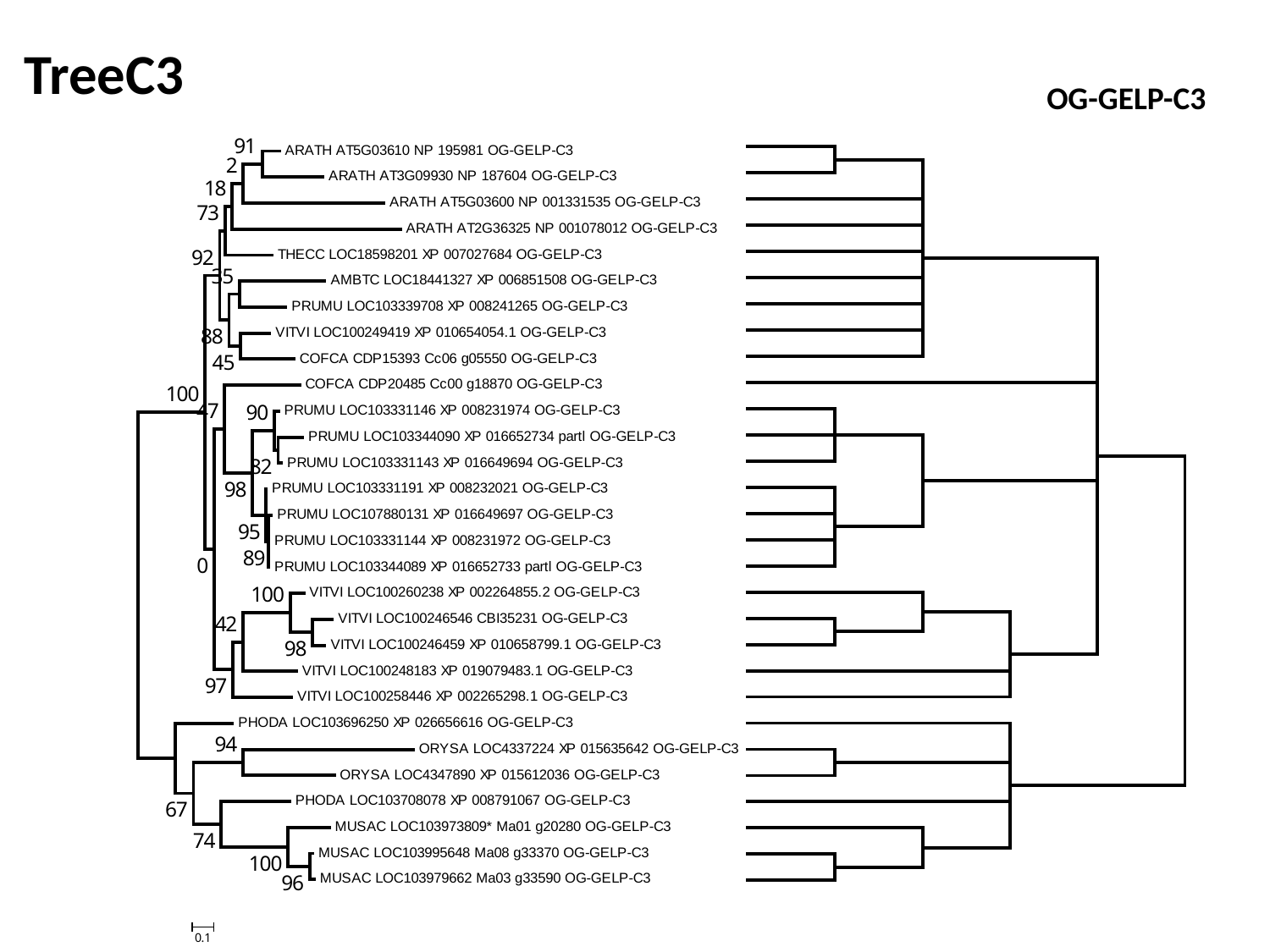

TreeC3
OG-GELP-C3

## Slide 12
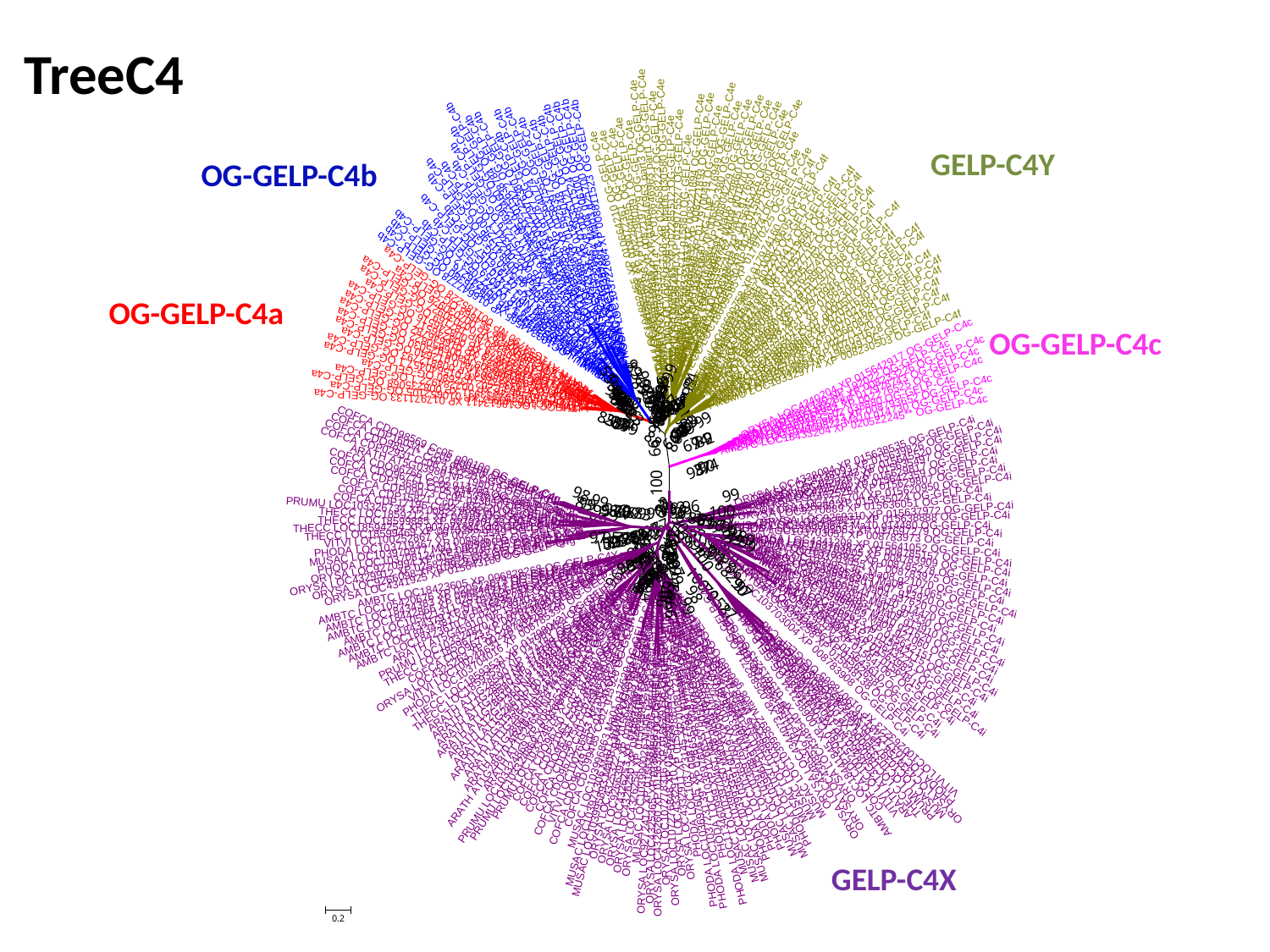

TreeC4
GELP-C4Y
OG-GELP-C4b
OG-GELP-C4a
OG-GELP-C4c
GELP-C4X

## Slide 13
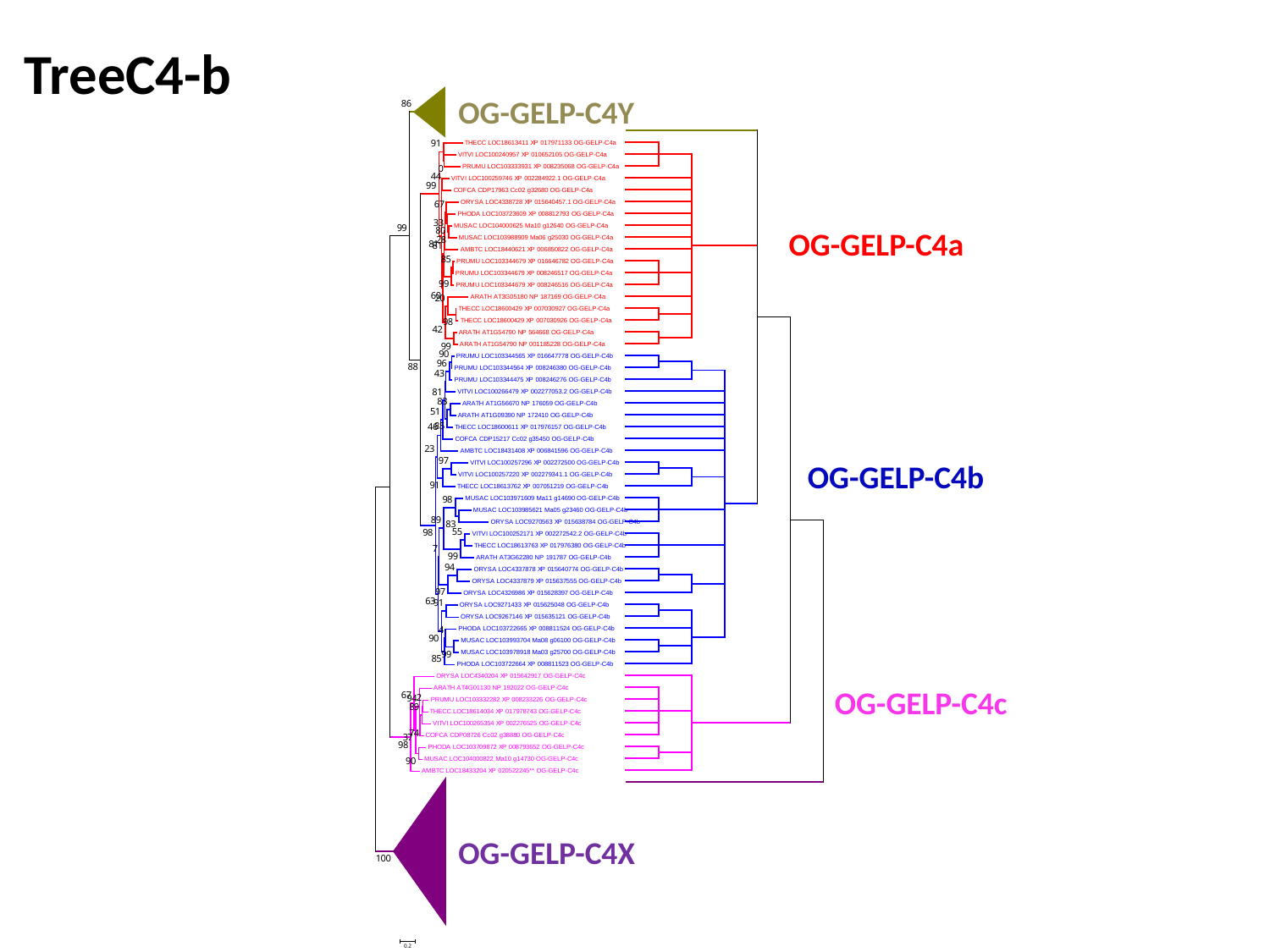

TreeC4-b
OG-GELP-C4Y
OG-GELP-C4a
OG-GELP-C4b
OG-GELP-C4c
OG-GELP-C4X

## Slide 14
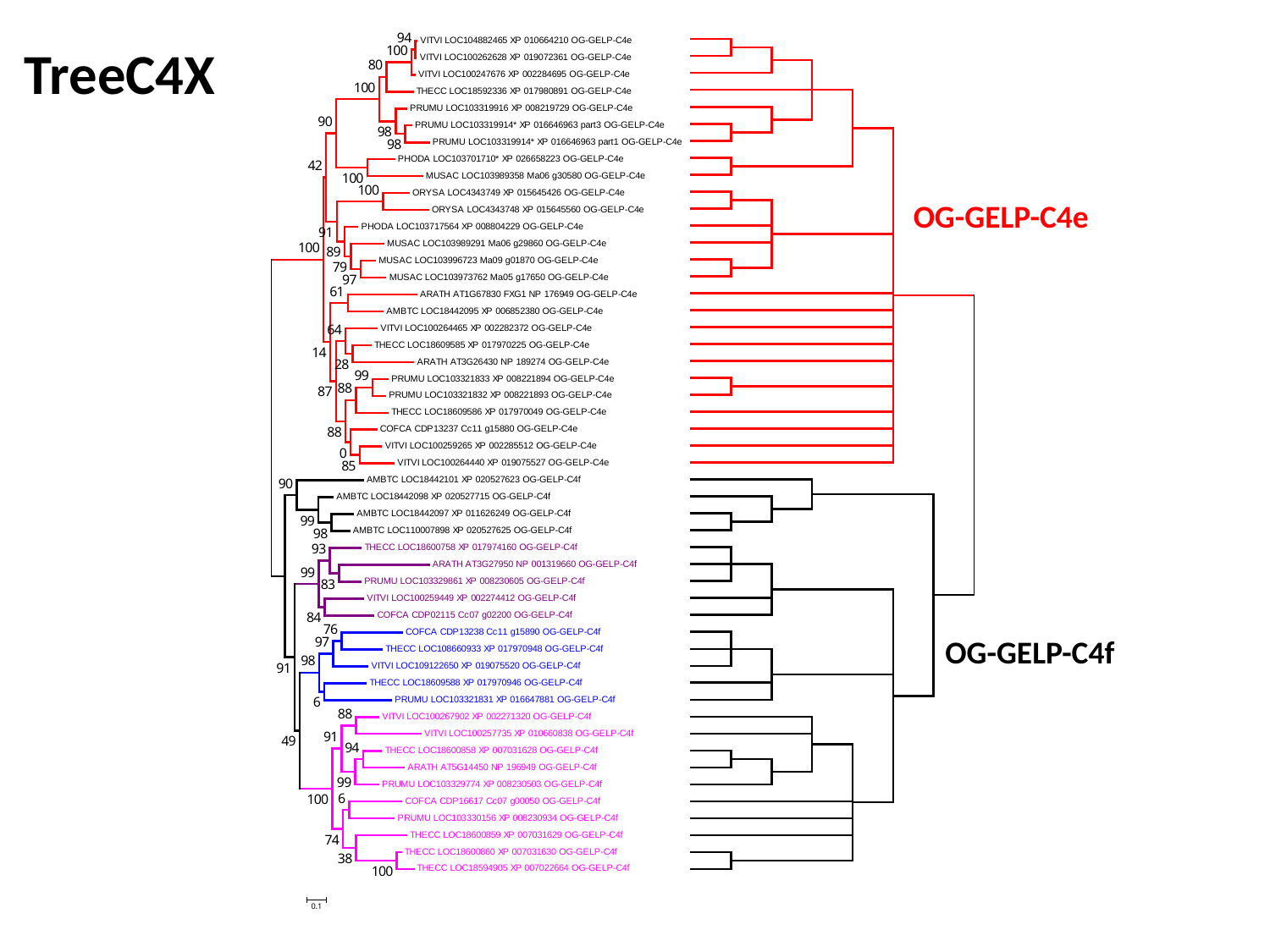

TreeC4X
OG-GELP-C4e
OG-GELP-C4f

## Slide 15
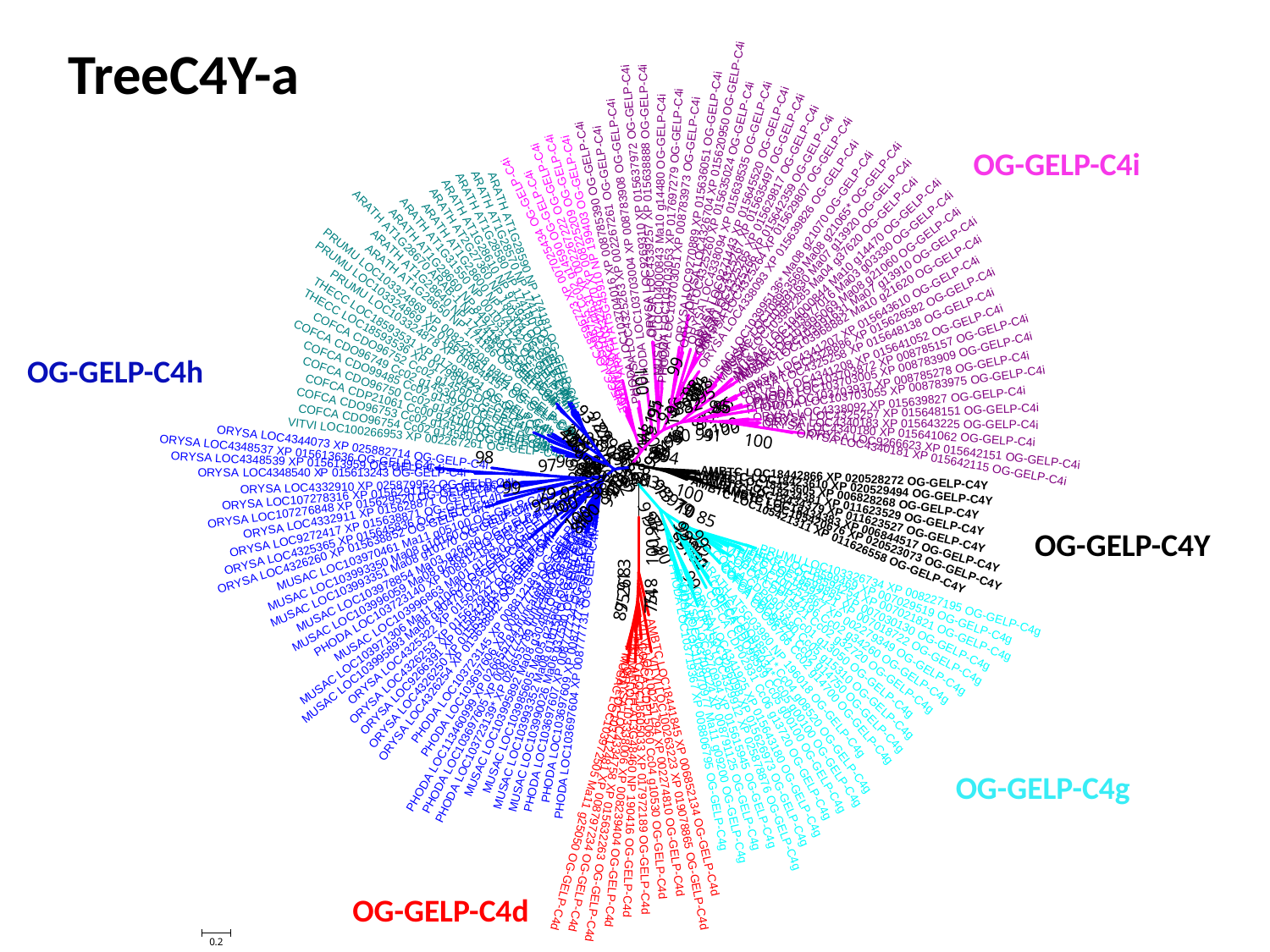

TreeC4Y-a
OG-GELP-C4i
OG-GELP-C4h
OG-GELP-C4Y
OG-GELP-C4g
OG-GELP-C4d

## Slide 16
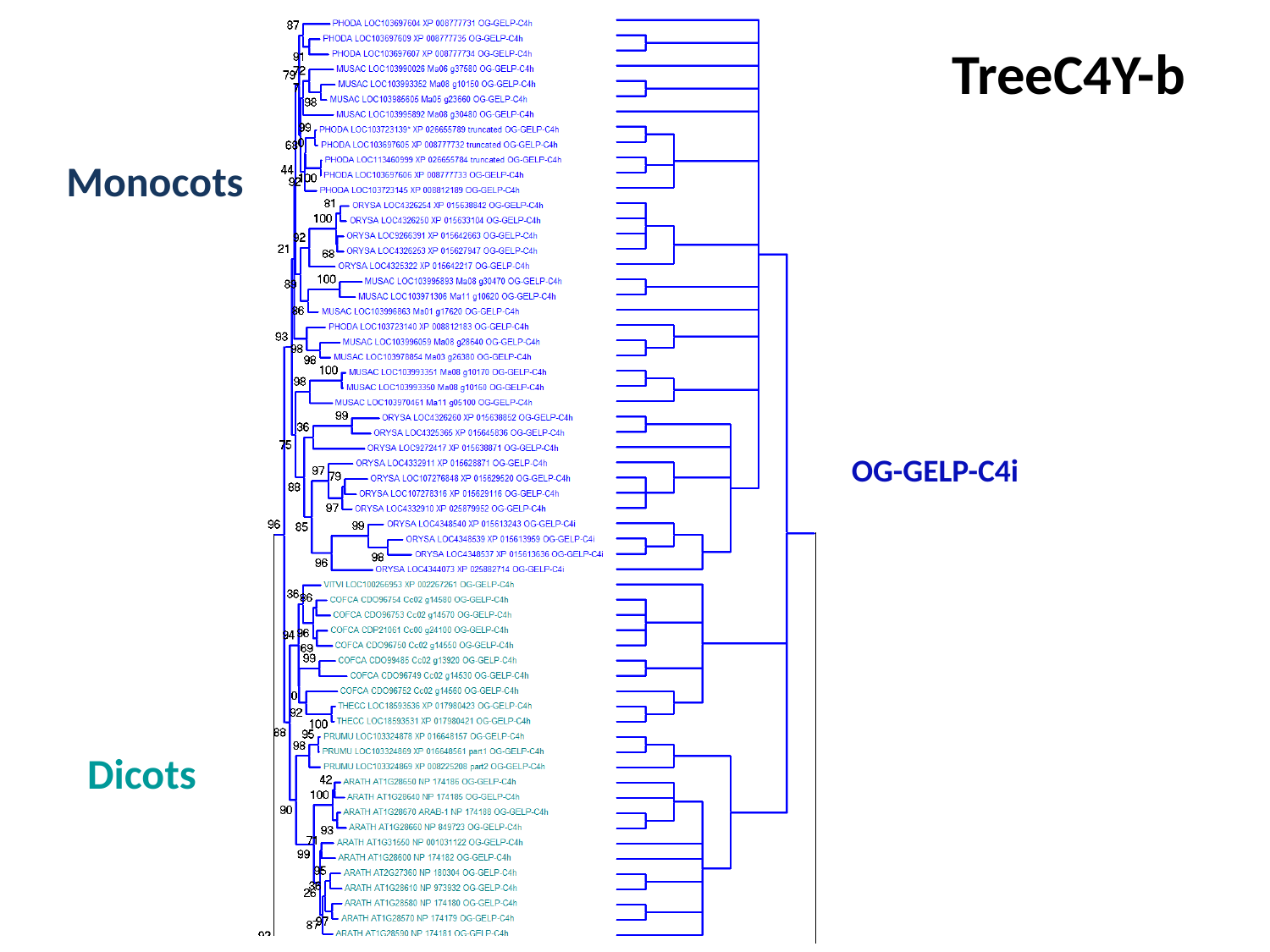

TreeC4Y-b
Monocots
OG-GELP-C4i
Dicots

## Slide 17
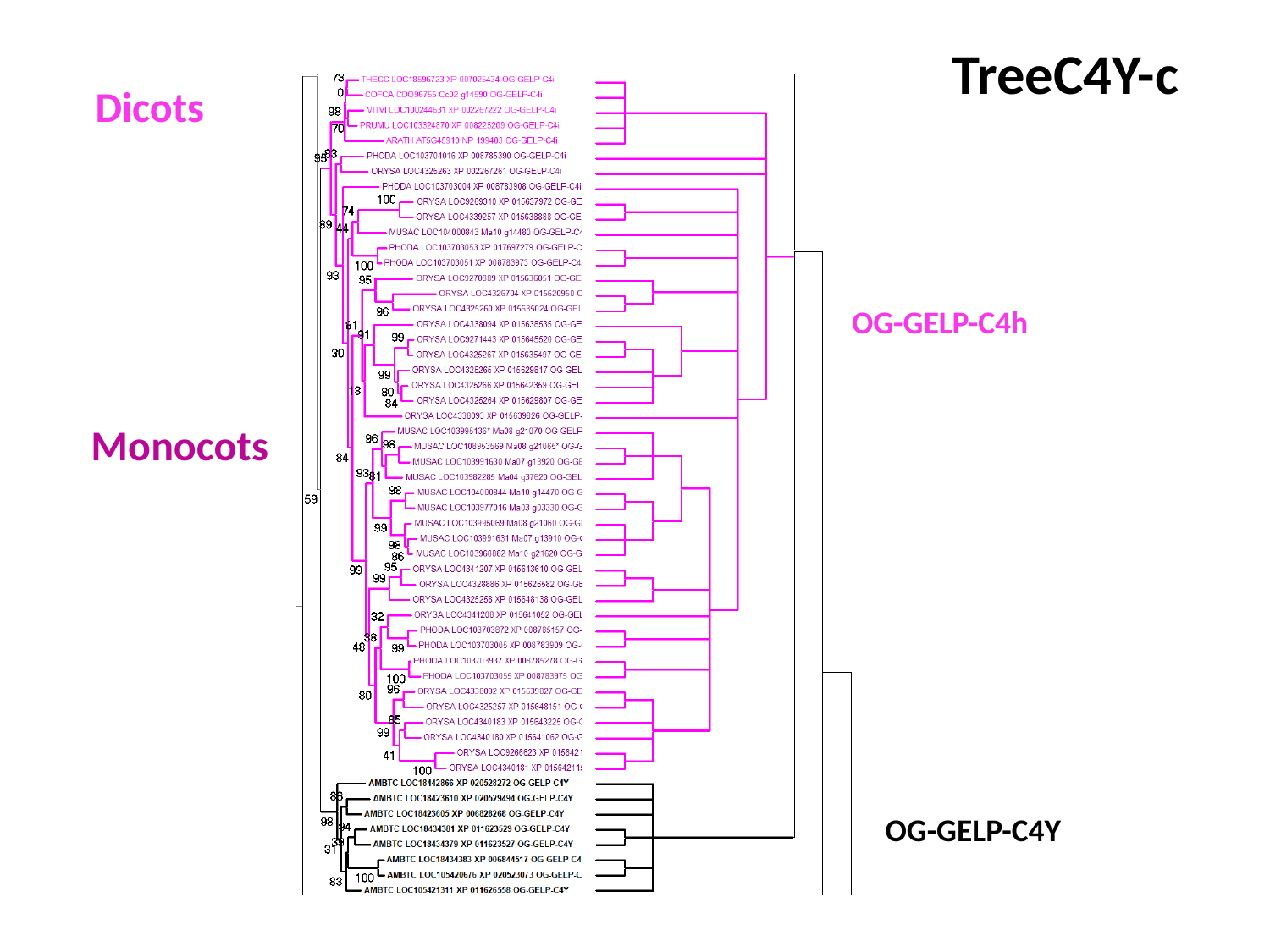

TreeC4Y-c
Dicots
OG-GELP-C4h
Monocots
OG-GELP-C4Y

## Slide 18
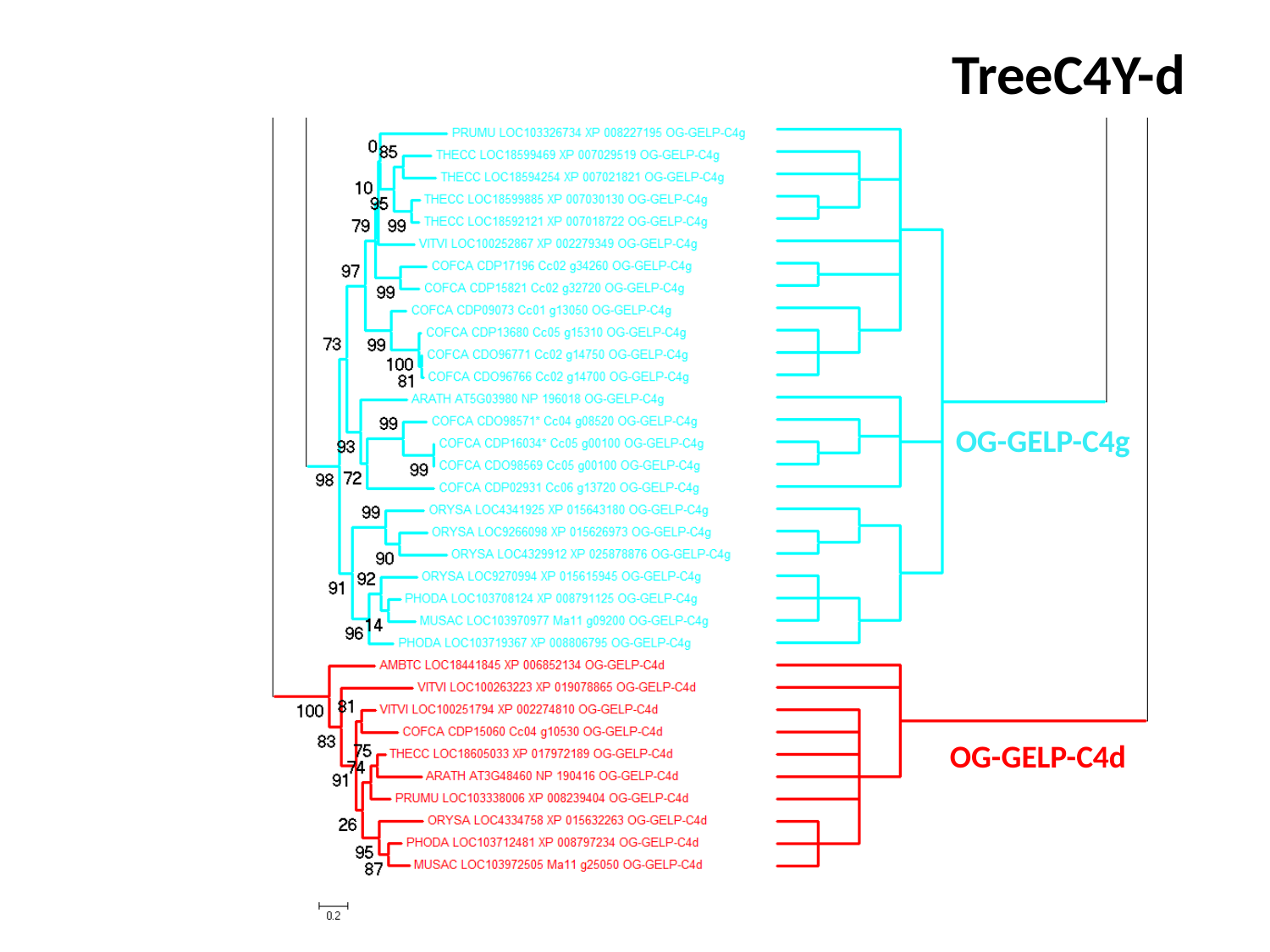

TreeC4Y-d
OG-GELP-C4g
OG-GELP-C4d

## Slide 19
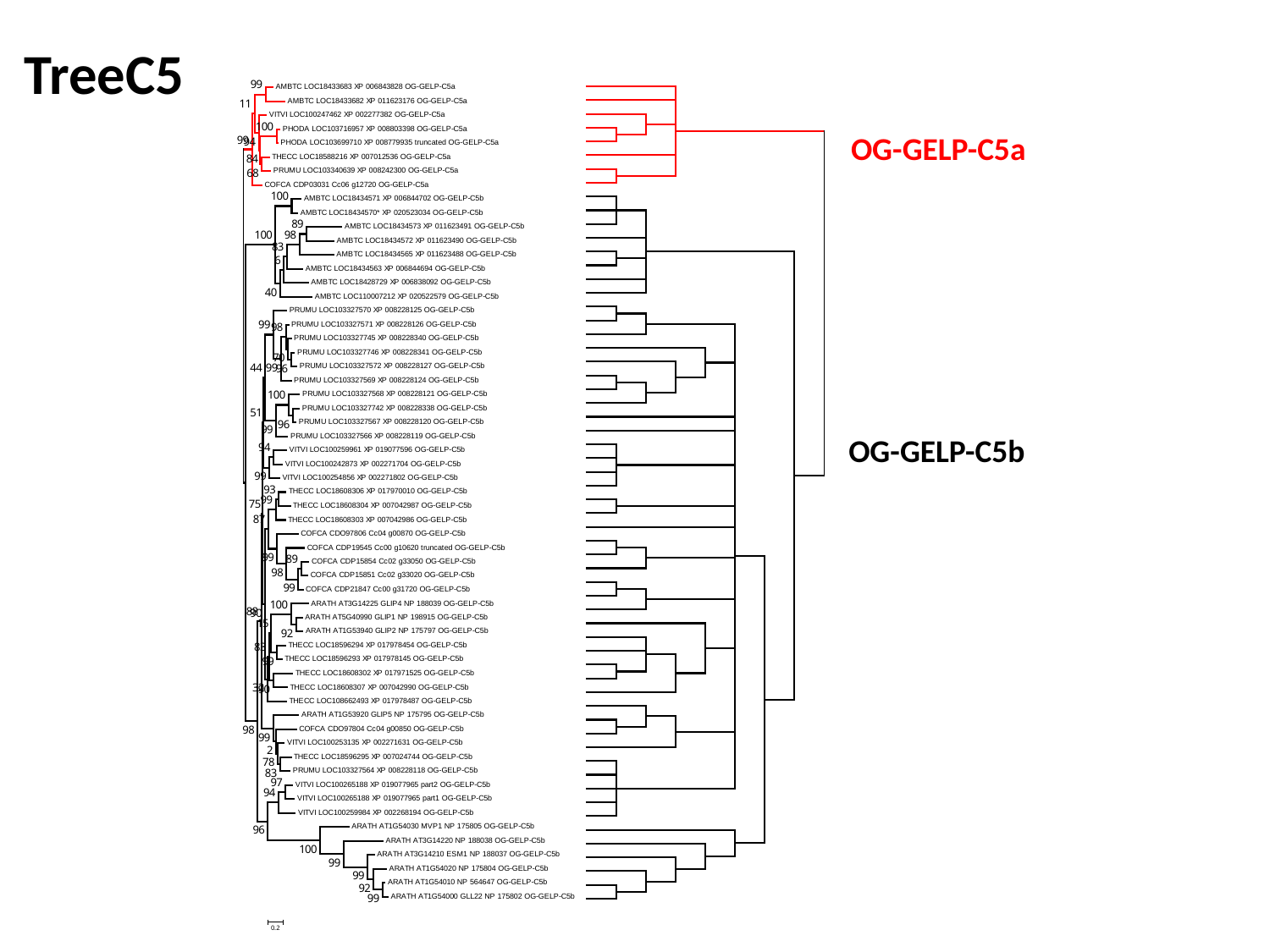

TreeC5
OG-GELP-C5a
OG-GELP-C5b

## Slide 20
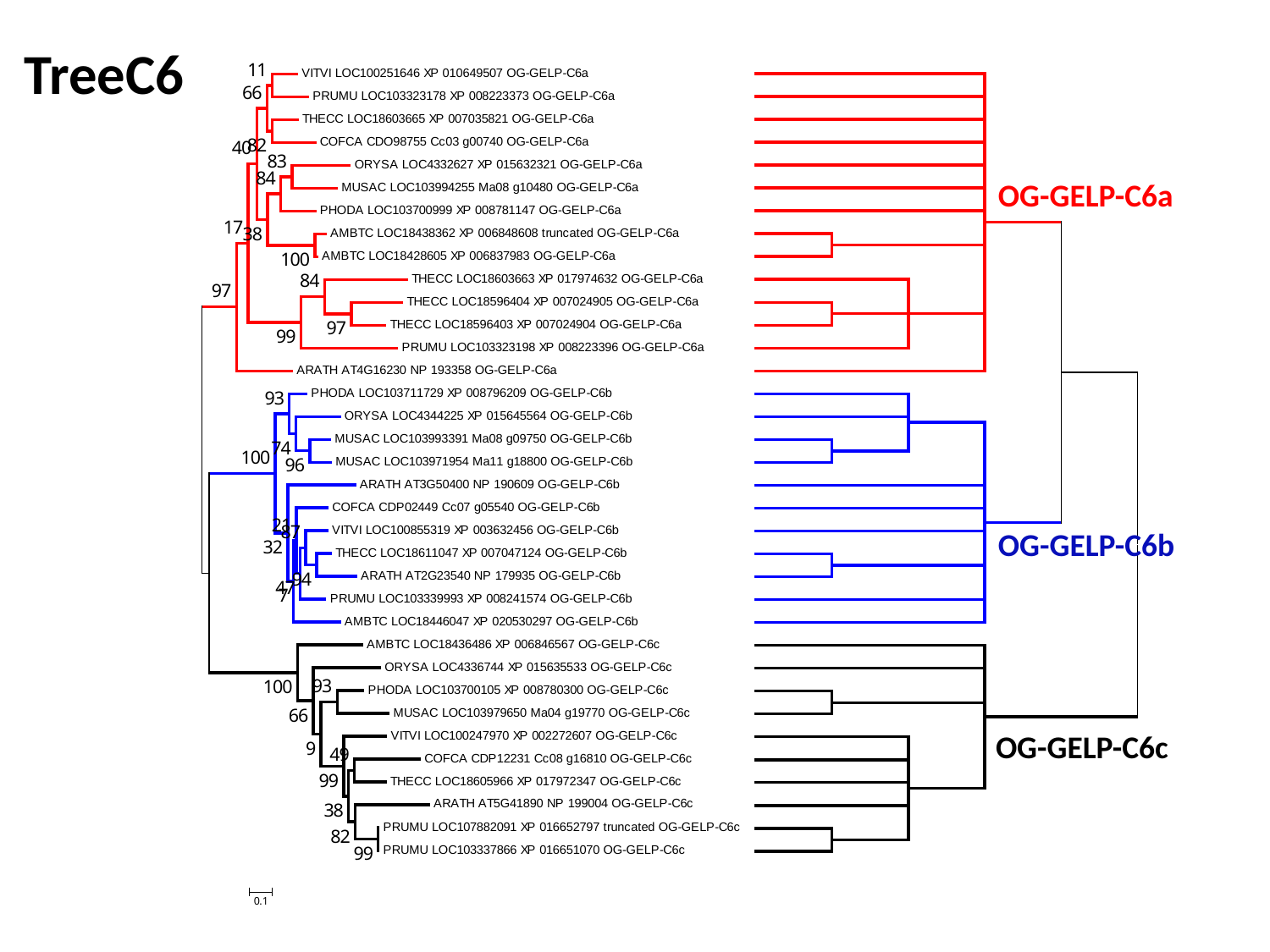

TreeC6
OG-GELP-C6a
OG-GELP-C6b
OG-GELP-C6c

## Slide 21
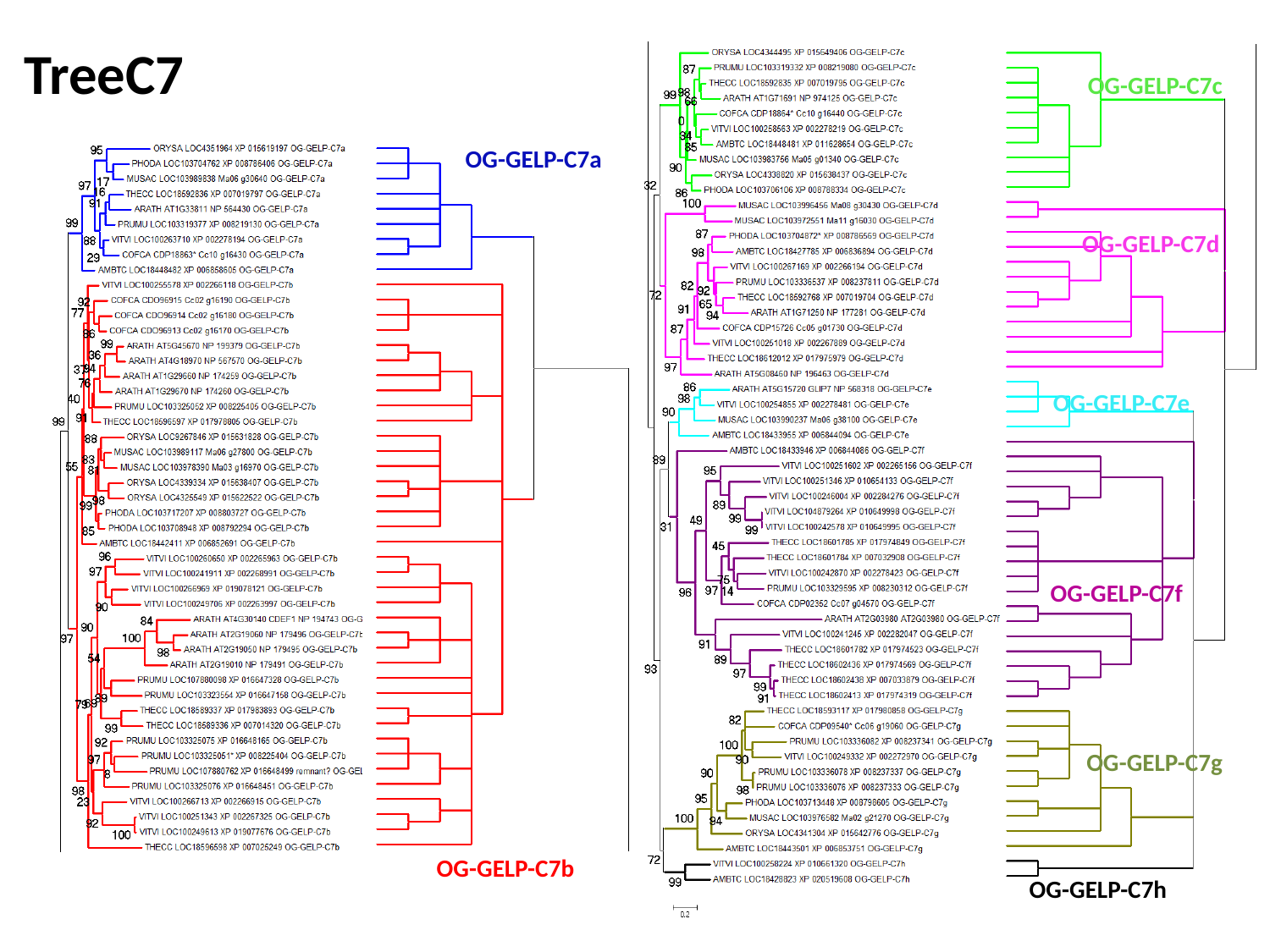

TreeC7
OG-GELP-C7c
OG-GELP-C7a
OG-GELP-C7d
OG-GELP-C7e
OG-GELP-C7f
OG-GELP-C7g
OG-GELP-C7b
OG-GELP-C7h

## Slide 22
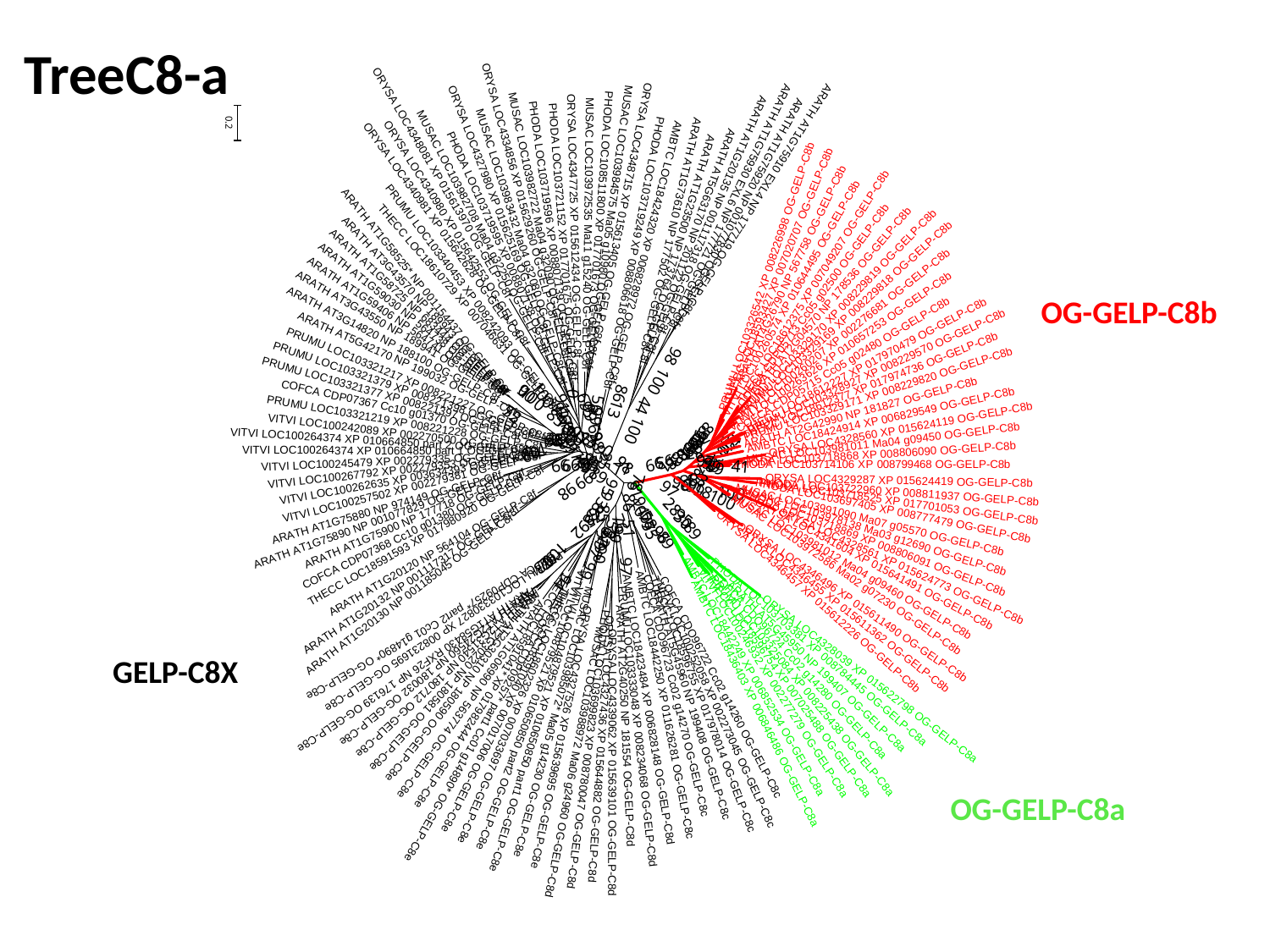

TreeC8-a
OG-GELP-C8b
GELP-C8X
OG-GELP-C8a

## Slide 23
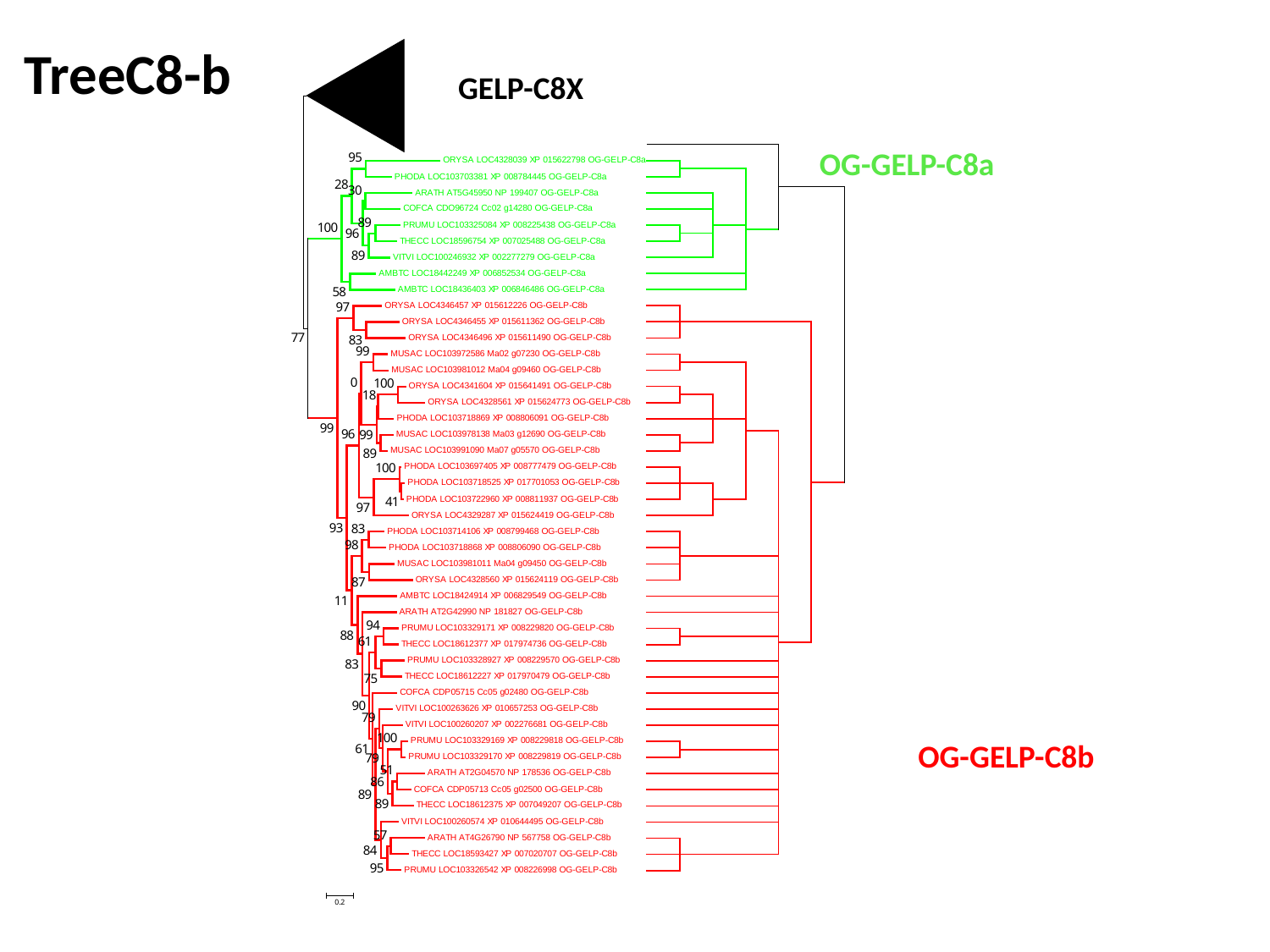

TreeC8-b
GELP-C8X
OG-GELP-C8a
OG-GELP-C8b

## Slide 24
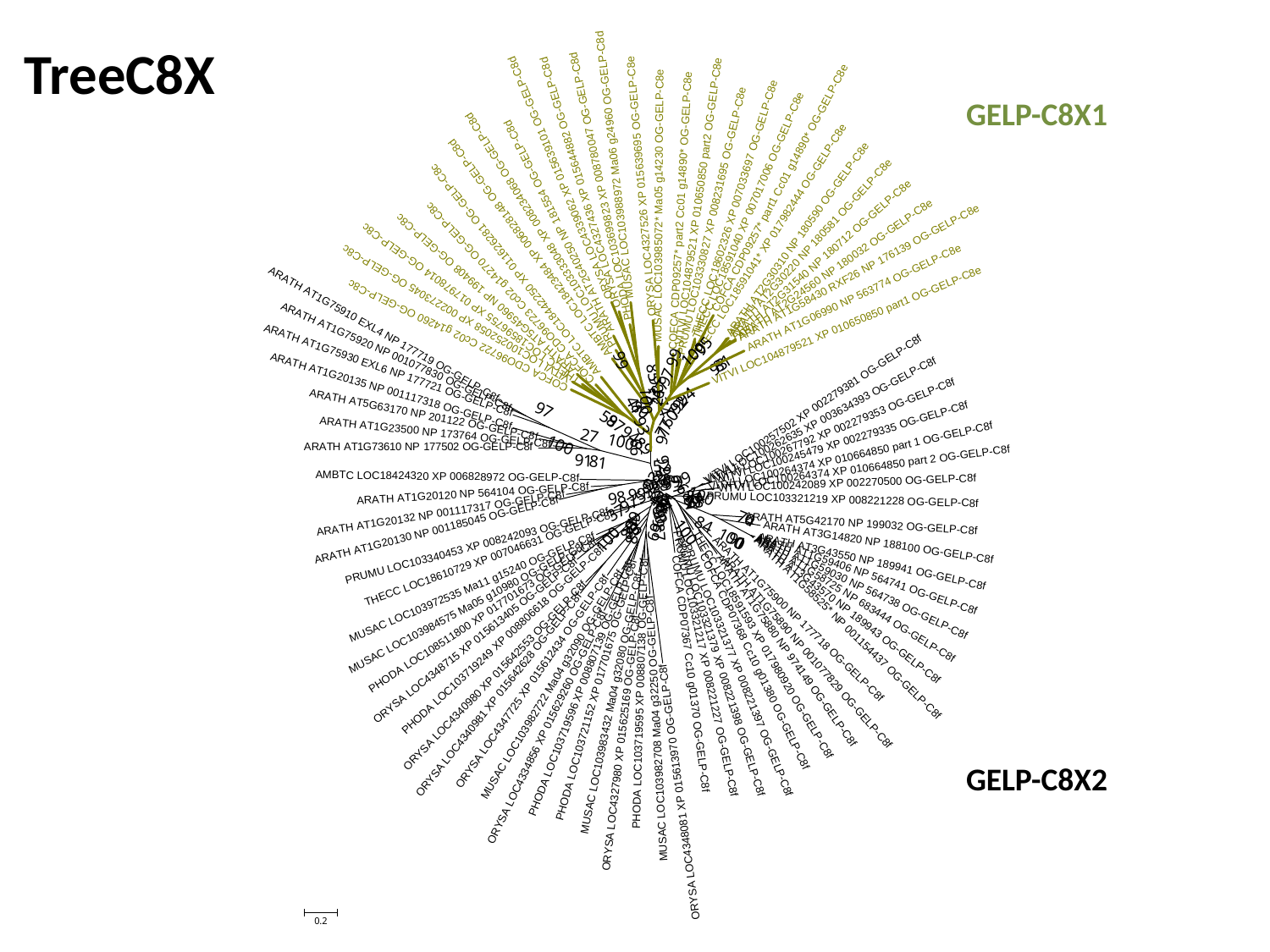

TreeC8X
GELP-C8X1
GELP-C8X2

## Slide 25
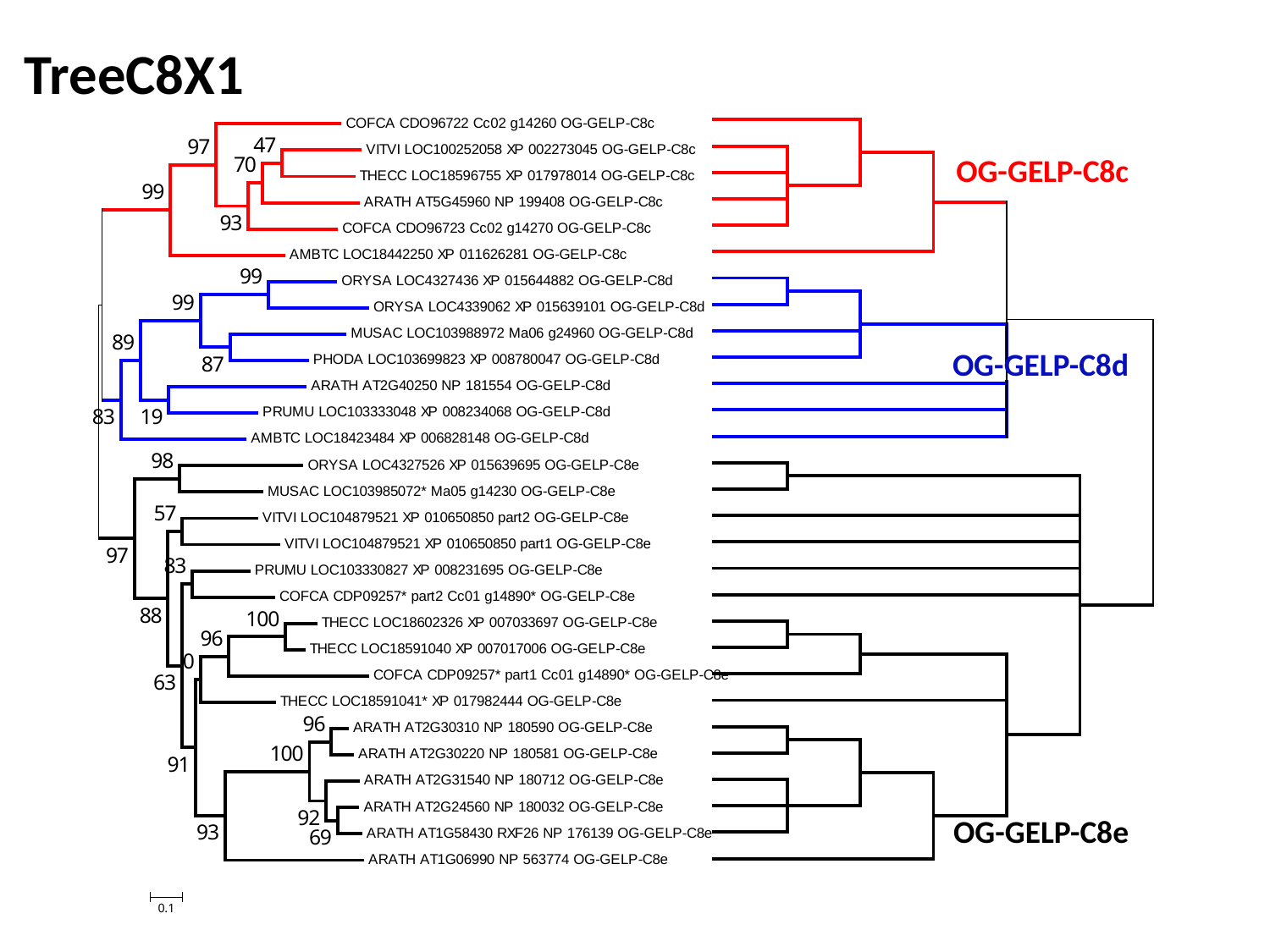

TreeC8X1
OG-GELP-C8c
OG-GELP-C8d
OG-GELP-C8e

## Slide 26
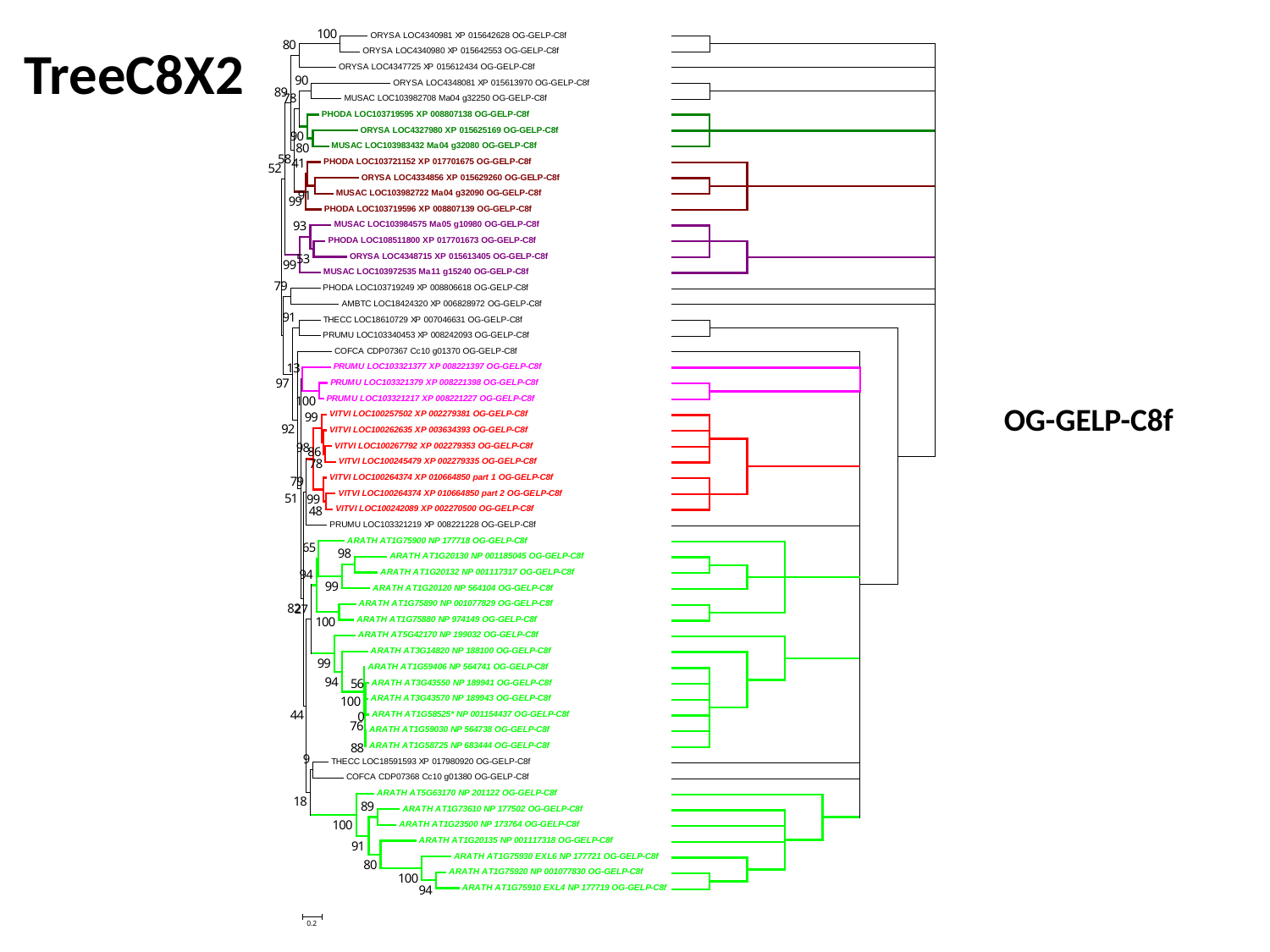

TreeC8X2
OG-GELP-C8f

## Slide 27
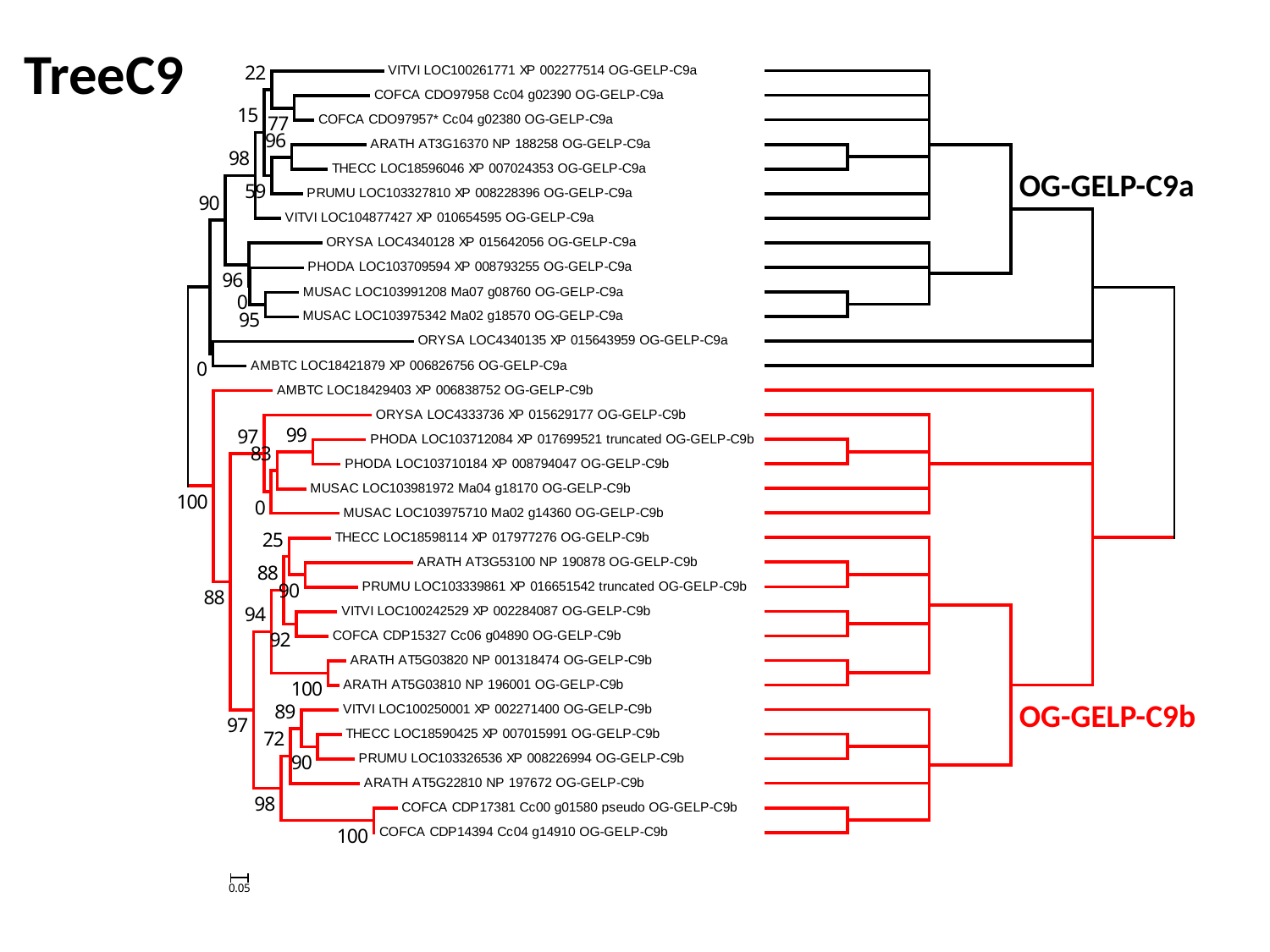

TreeC9
OG-GELP-C9a
OG-GELP-C9b

## Slide 28
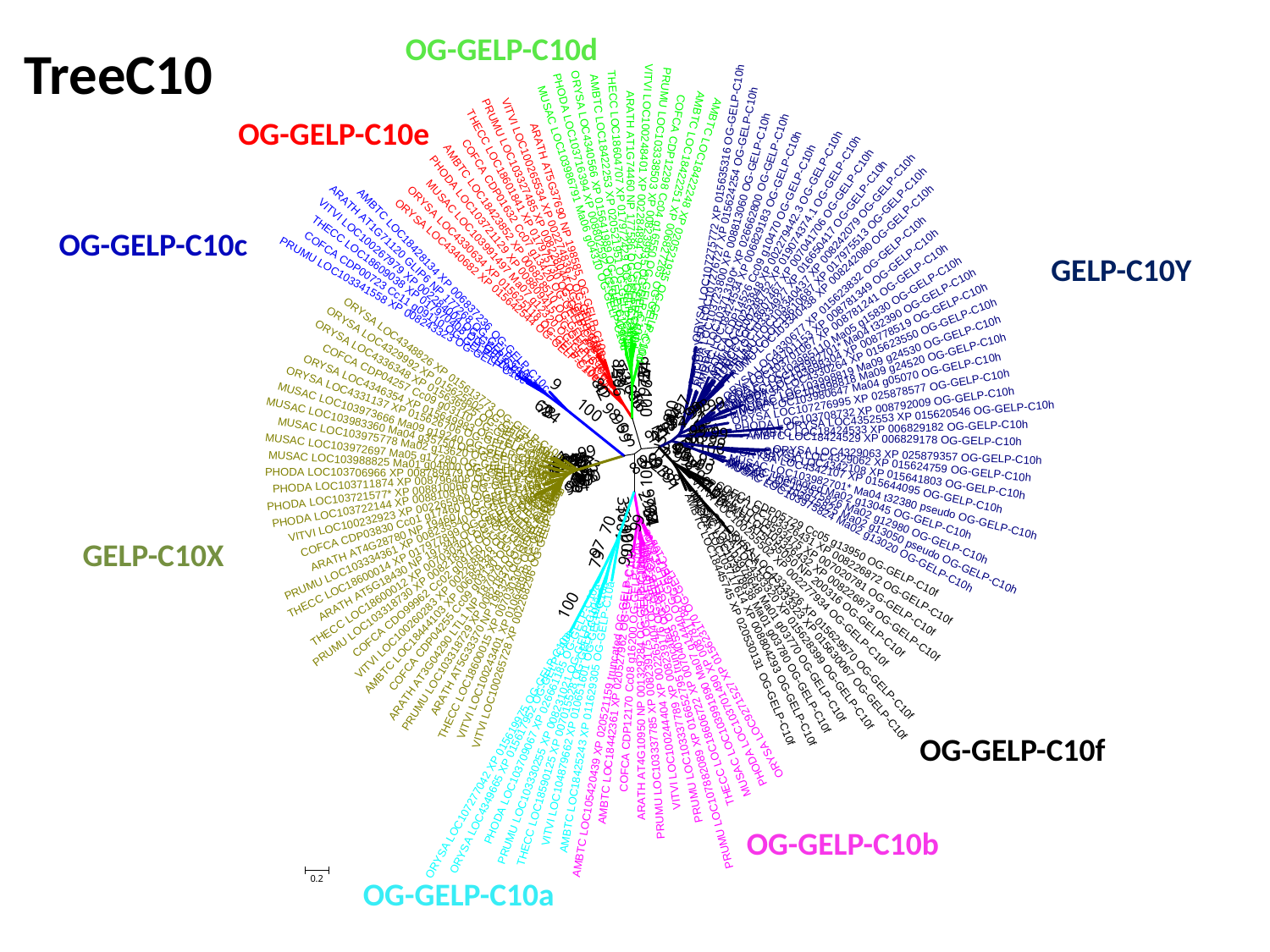

OG-GELP-C10d
TreeC10
OG-GELP-C10e
OG-GELP-C10c
GELP-C10Y
GELP-C10X
OG-GELP-C10f
OG-GELP-C10b
OG-GELP-C10a

## Slide 29
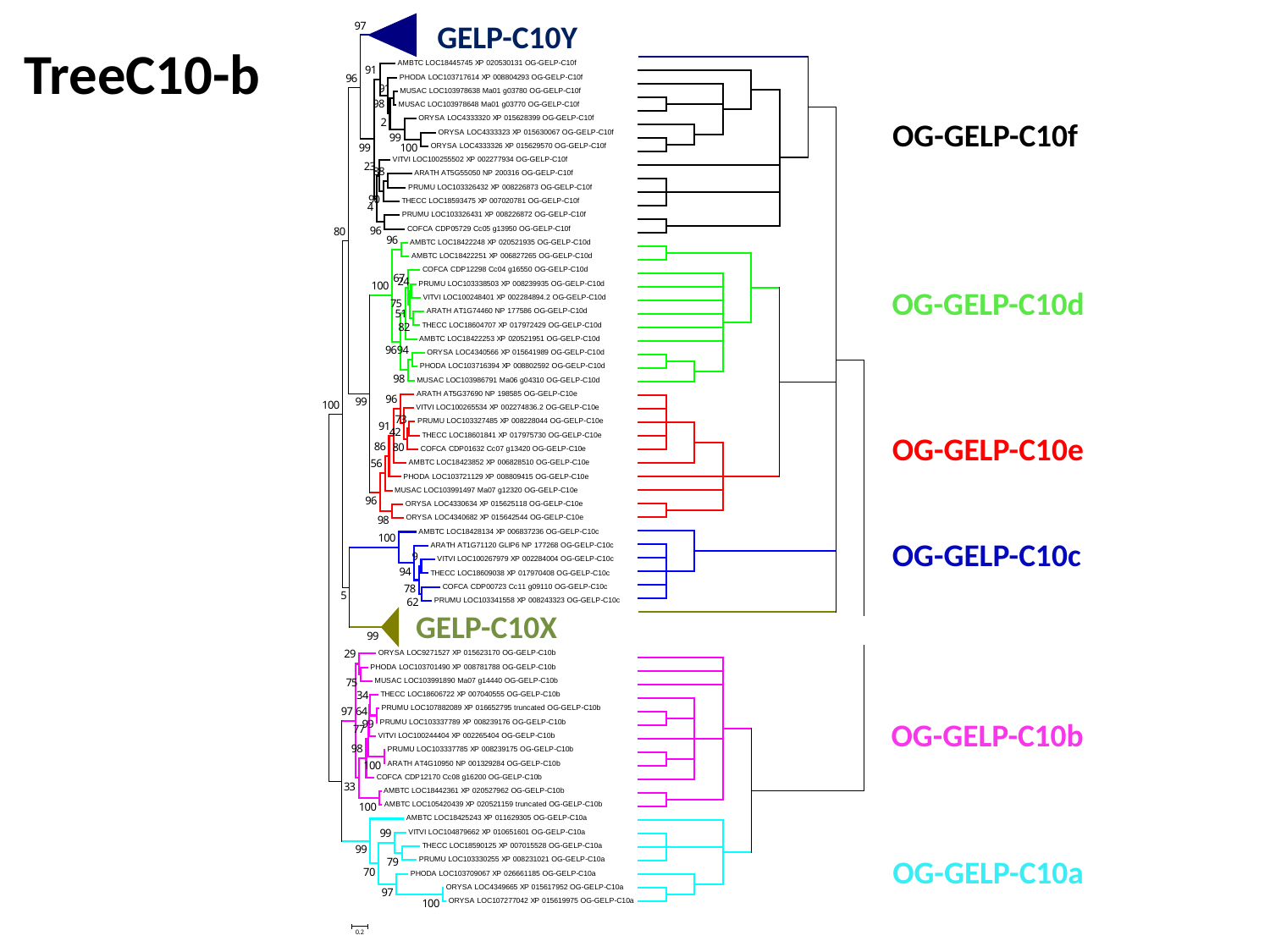

GELP-C10Y
TreeC10-b
OG-GELP-C10f
OG-GELP-C10d
OG-GELP-C10e
OG-GELP-C10c
GELP-C10X
OG-GELP-C10b
OG-GELP-C10a

## Slide 30
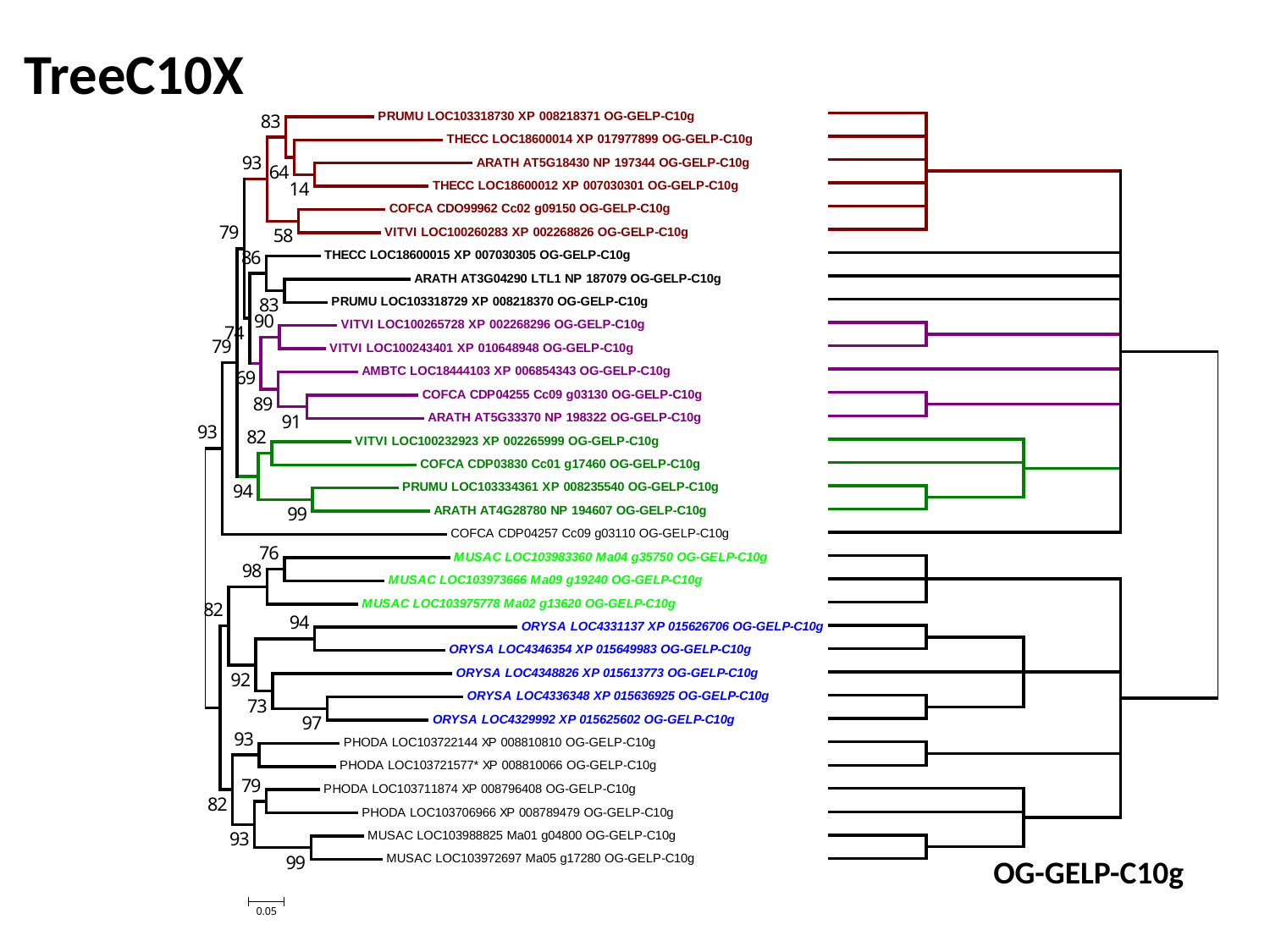

TreeC10X
OG-GELP-C10g

## Slide 31
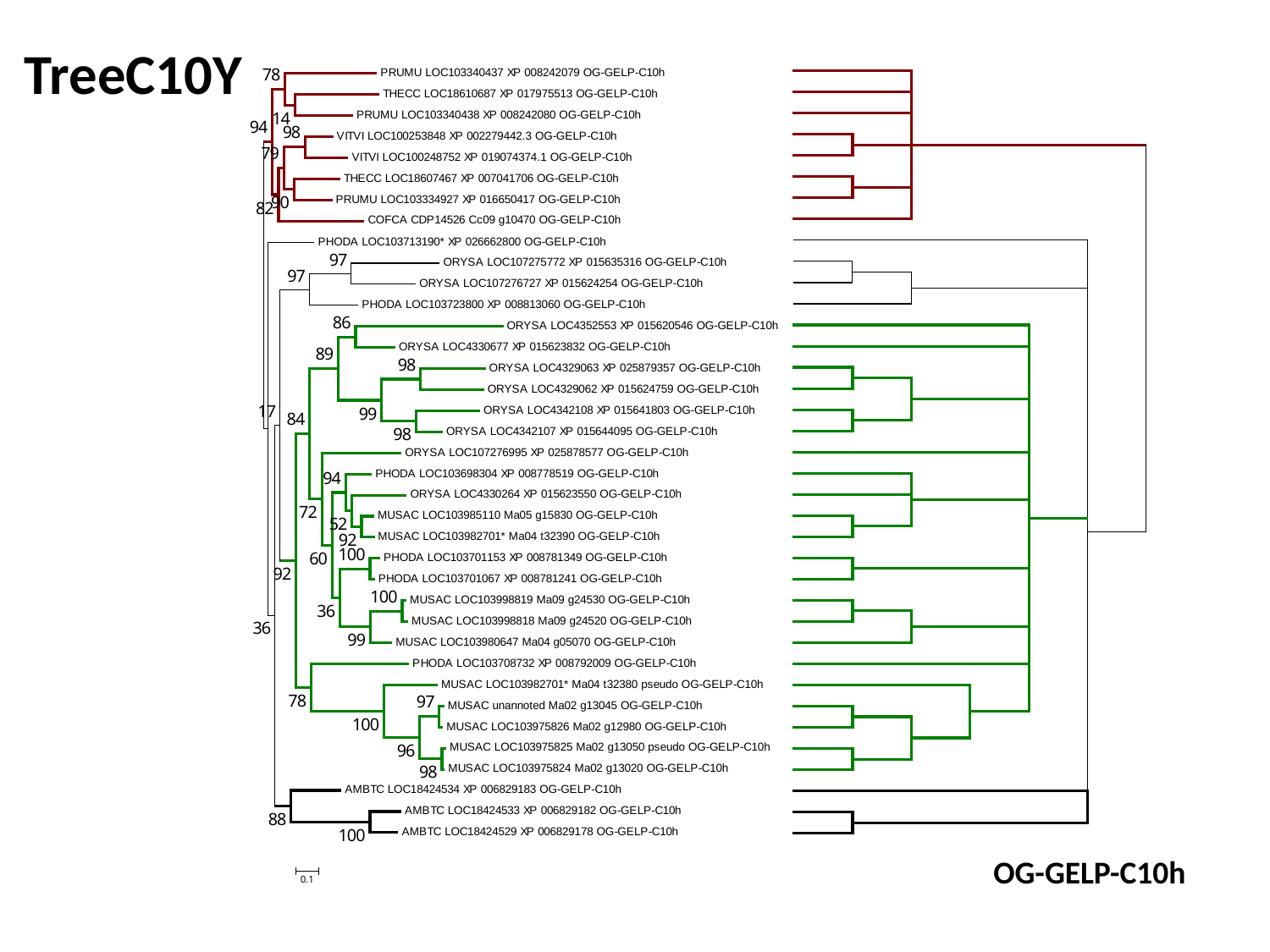

TreeC10Y
OG-GELP-C10h
